# Inhibition of the SR Protein-Phosphorylating CLK Kinases of *Plasmodium falciparum* Impairs Blood Stage Replication and Malaria Transmission

**DOI:** 10.1371/journal.pone.0105732

**Published:** 2014-09-04

**Authors:** Selina Kern, Shruti Agarwal, Kilian Huber, André P. Gehring, Benjamin Strödke, Christine C. Wirth, Thomas Brügl, Liliane Onambele Abodo, Thomas Dandekar, Christian Doerig, Rainer Fischer, Andrew B. Tobin, Mahmood M. Alam, Franz Bracher, Gabriele Pradel

**Affiliations:** 1 Research Center for Infectious Diseases, University of Würzburg, Würzburg, Germany; 2 Institute of Molecular Biotechnology, RWTH Aachen University, Aachen, Germany; 3 Department of Pharmacy – Center for Drug Research, Ludwig-Maximillians University, Munich, Germany; 4 Bioinformatics, Biocenter, University of Würzburg, Würzburg, Germany; 5 INSERM U609, Global Health Institute, Ecole Polytechnique Fédérale de Lausanne (EPFL), Lausanne, Switzerland; 6 Department of Microbiology, Monash University, Clayton, Victoria, Australia; 7 Department of Cell Physiology and Pharmacology, MRC Toxicology Unit, University of Leicester, Leicester, United Kingdom; Centro de Pesquisa Rene Rachou/Fundação Oswaldo Cruz (Fiocruz-Minas), Brazil

## Abstract

Cyclin-dependent kinase-like kinases (CLKs) are dual specificity protein kinases that phosphorylate Serine/Arginine-rich (SR) proteins involved in pre-mRNA processing. Four CLKs, termed PfCLK-1-4, can be identified in the human malaria parasite *Plasmodium falciparum*, which show homology with the yeast SR protein kinase Sky1p. The four PfCLKs are present in the nucleus and cytoplasm of the asexual blood stages and of gametocytes, sexual precursor cells crucial for malaria parasite transmission from humans to mosquitoes. We identified three plasmodial SR proteins, PfSRSF12, PfSFRS4 and PfSF-1, which are predominantly present in the nucleus of blood stage trophozoites, PfSRSF12 and PfSF-1 are further detectable in the nucleus of gametocytes. We found that recombinantly expressed SR proteins comprising the Arginine/Serine (RS)-rich domains were phosphorylated by the four PfCLKs in *in vitro* kinase assays, while a recombinant PfSF-1 peptide lacking the RS-rich domain was not phosphorylated. Since it was hitherto not possible to knock-out the *pfclk* genes by conventional gene disruption, we aimed at chemical knock-outs for phenotype analysis. We identified five human CLK inhibitors, belonging to the oxo-β-carbolines and aminopyrimidines, as well as the antiseptic chlorhexidine as PfCLK-targeting compounds. The six inhibitors block *P. falciparum* blood stage replication in the low micromolar to nanomolar range by preventing the trophozoite-to-schizont transformation. In addition, the inhibitors impair gametocyte maturation and gametogenesis in *in vitro* assays. The combined data show that the four PfCLKs are involved in phosphorylation of SR proteins with essential functions for the blood and sexual stages of the malaria parasite, thus pointing to the kinases as promising targets for antimalarial and transmission blocking drugs.

## Introduction

The protozoan parasite *Plasmodium falciparum* is responsible for more than 600,000 fatal cases caused by the tropical disease malaria per annum [1]. During life cycle progression from humans to mosquitoes, *P. falciparum* switches between stages with high replication rates and ones arrested in their cell cycle and also passes through a phase of sexual reproduction. These rapid transformations require fine-tuned mechanisms of gene expression, and the importance of post-transcriptional regulation of gene expression in *Plasmodium* parasites has previously been highlighted [2]. These include the alternative splicing (AS) of pre-mRNA, enabling the parasite to express functionally different protein isoforms. Two genome-wide studies implied that more than 200 AS events occur during blood stage replication of *P. falciparum* [3], [4].

AS involves multiple auxiliary factors that control splice site selection, spliceosome assembly as well as the splicing reaction [5], [6]. One group of proteins regulating the selection of alternatively spliced exonic or intronic pre-mRNA sequences is that of Serine/Arginine-rich (SR) proteins [5]. SR proteins usually contain a RNA recognition motif and an Arginine/Serine-rich (RS) domain required for protein-protein interactions during splicing. SR proteins are phosphorylated by several protein kinase families, including the cyclin-dependent kinase-like kinases (CLKs) [7], [8].

The genome of *P. falciparum* encodes four members of the CLK family, which were previously termed PfCLK-1-4 [9]–[11]. For PfCLK-1 (originally described as LAMMER kinase) [12] and PfCLK-2 homologies with the yeast SR protein kinase Sky1p were shown [11]. Both kinases are expressed in the *P. falciparum* blood stages and phosphorylate a number of substrates *in vitro*, including the Sky1p substrate, SR protein Npl3p, and the plasmodial alternative splicing factor PfASF-1 [11]. Similarly, PfCLK-4 (also known as SRPK1) is expressed in the blood stages of *P. falciparum*, where it phosphorylates the plasmodial protein SR1 [13], an AS factor required for parasite proliferation [14]. Previous reverse genetics studies indicated an important role of the four PfCLKs for completion of the asexual replication cycle, since disruption of the respective genes was not achievable, while the loci were amenable to a modification that does not cause loss-of-function [11], [15].

Here we aimed to investigate the involvement of the four PfCLKs in the phosphorylation of plasmodial SR proteins and to determine the crucial role of the kinases for the blood and transmission stages of *P. falciparum* via chemical knock-outs using a variety of newly identified CLK inhibitors.

## Materials and Methods

### Gene IDs and data analysis

**Figure 1:**
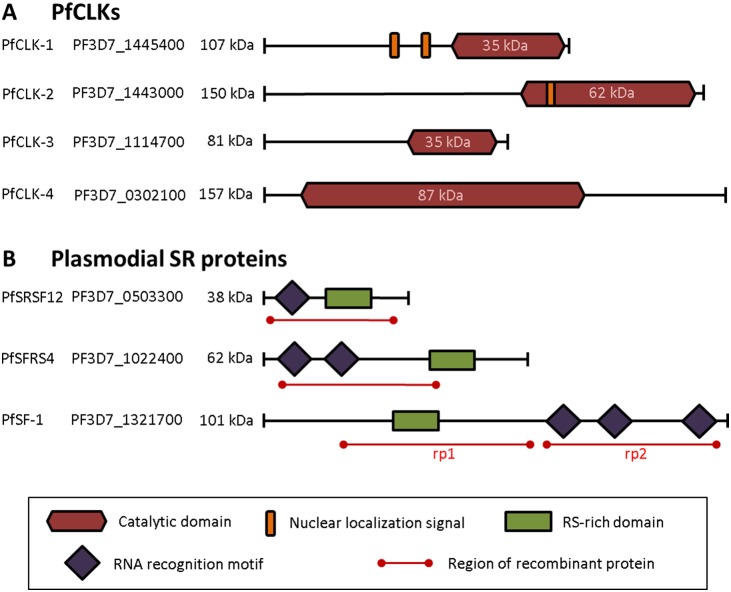
Schematic of the plasmodial PfCLKs and SR proteins. A. Domain structures of the PfCLKs. B. Domain structures of the plasmodial SR proteins investigated in this study.

The following PlasmoDB gene identifiers (plasmodb.org; previous IDs set in brackets) [16], [17] are assigned to the CLKs and SR proteins investigated in this study (shown in Fig. 1): PfCLK-1, PF3D7_1445400 (PF14_0431); PfCLK-2, PF3D7_1443000 (PF14_0408); PfCLK-3, PF3D7_1114700 (PF11_0156); PfCLK-4, PF3D7_0302100 (PFC0105w); PfPKRP, PF3D7_0311400 (PFC0485w); PfSFRS4, PF3D7_1022400 (PF10_0217); PfSRSF12, PF3D7_0503300 (PFE0160c); PfSF-1, PF3D7_1321700 (MAL13P1.120).

### Bioinformatics

The following computer programs and databases were used for the *in silico* studies: For gene sequence annotation, PlasmoDB (www.plasmodb.org) [16], [17], the SMART program (www.smart.embl-heidelberg.de) [18], [19] and NCBI sequence analysis software and databanks [20] were used. Multiple sequence alignment involved programs ClustalW (www.ebi.ac.uk/clustalw) [21] and Clone Manager 9, and formatting of multiple sequence alignments was pursued according to standard methods (espript.ibcp.fr).

### CLK inhibitors

**Figure 2:**
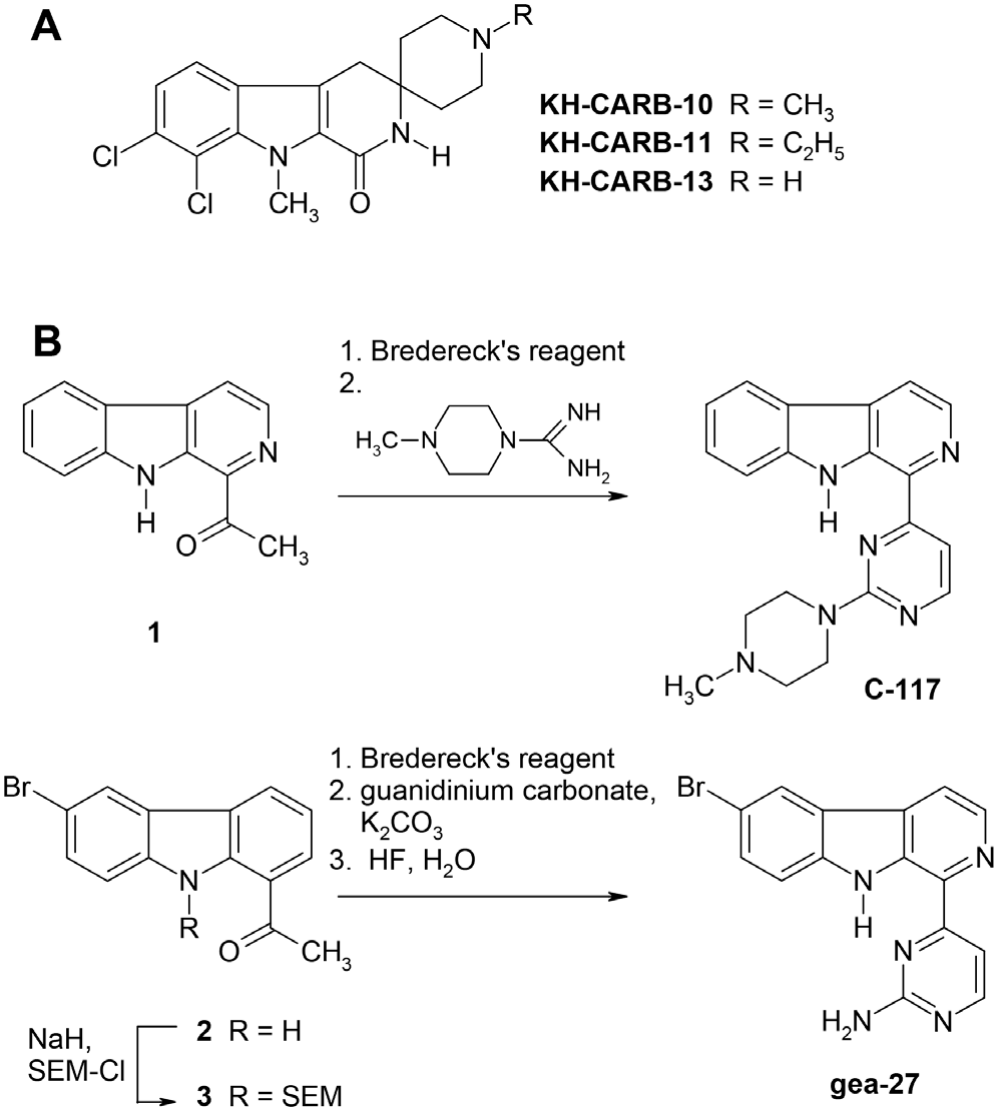
Chemical structures of CLK inhibitors. A. Structures of the spiropiperidino-β-carbolines KH-CARB-10, KH-CARB-11, and KH-CARB-13xHCl. B. Synthesis of the aminopyrimidyl β-carboline C-117 and the aminopyrimidyl carbazole gea-27.

Chlorhexidine (CHX) was purchased from Sigma-Aldrich. The spiropiperidino-β-carbolines KH-CARB-10, KH-CARB-11, and KH-CARB-13xHCl were prepared as described previously (Fig. 2A) [22]. The aminopyrimidyl β-carboline C-117 and the aminopyrimidyl carbazole gea-27 were prepared starting from known methyl ketones as precursors (Fig. 2B). In short, treatment of 1-acetyl-β-carboline (1; see Fig. 2B) [23] with tert-butoxy-bis(dimethylamino)methane (Bredereck’s reagent) in refluxing dimethylformamide, followed by addition of 4-methylpiperazine-1-carboxamidinium sulfate and potassium carbonate gave the target compound C-117 in good yield in one single operation [24]. For the synthesis of gea-27 the acetylcarbazole (2) [25] was protected at the pyrrole nitrogen with the SEM (2-(trimethylsilyl)-ethoxymethyl) group to give (3), then heated with Bredereck’s reagent and subsequently with guanidinium carbonate and potassium carbonate. The resulting aminopyrimidine intermediate was deprotected with HF to give the target compound. Syntheses of C-117 and gea-27 are described in detail in (Methods S1). All inhibitors were prepared as 100 mM stock solutions in dimethyl sulfoxide (DMSO).

### Parasite culture

Asexual blood stage parasites and gametocytes of the *P. falciparum* NF54 [26] isolate and asexual blood stage parasites of the *P. falciparum* strains 3D7 [27] and F12 [28] were cultivated in human erythrocytes *in vitro* as described [29]–[31]. The following parasite lines were obtained through the MR4 as part of the BEI Resources Repository, NIAID, NIH: *Plasmodium falciparum* NF54, MRA-1000, deposited by M Dowler, Walter Reed Army Institute of Research and *Plasmodium falciparum* 3D7, MRA-102, deposited by DJ Carucci. Parasite line F12 was kindly provided by Pietro Alano, Istituto Superiore di Sanità, Rome. Human A^+^ erythrocyte sediment and serum were purchased from the University Hospital Aachen, Germany (PO no. DKG-NT 9748). The erythrocyte and sera samples were pooled and the donors remained anonymous; the work on human blood was approved by the ethics commission of RWTH Aachen University. RPMI medium 1640 (Gibco) was supplemented with either A^+^ human serum (for NF54 and F12) or 0.5% Albumax II (for 3D7; Invitrogen), hypoxanthine (Sigma-Aldrich) and gentamicin (Invitrogen) and cultures were maintained at 37°C in an atmosphere of 5% O_2_, 5% CO_2_, 90% N_2_. Gametogenesis was induced by incubating mature gametocyte cultures in 100 µM xanthurenic acid for 15 min at room temperature (RT) [32], [33]. For synchronization, parasite cultures with 3–4% ring stages were centrifuged to obtain the pellet, which was resuspended in five times pellet’s volume of 5% prewarmed sorbitol (AppliChem) in RPMI medium (Invitrogen) and incubated at RT for 10 min [34]. The cells were washed once with RPMI medium to remove the sorbitol, diluted to 5% vol. hematocrit and cultured as described above.

### Recombinant protein expression

Recombinant proteins were expressed as fusion proteins either with a glutathione S-transferase (GST)-tag using the pGEX 4T1 vector (Amersham Bioscience) or with a maltose binding protein (MaBP)-tag using the pIH-vector (kindly provided by Kim Williamson, Loyola University Chicago). Cloning into the pGEX4T1 vector was mediated by *Eco*RI/*Not*I restriction sites added at the ends of PCR-amplified gene fragments and cloning into the pIH-vector was mediated by *Eco*RI*/Sal*I restriction sites. Primers used for cloning are listed in (Table S1). Recombinant proteins were expressed in *E. coli* BL21 (DE3) RIL according to the manufacturer’s protocol (Stratagene). GST-fusion proteins were purified from bacterial extracts using glutathione-sepharose according to the manufacturer’s protocol (GE Healthcare). MaBP-tagged recombinant proteins were purified using amylose resin (New England Biolabs) as described previously [35] with following modifications of the procedure: pelleted bacteria were directly resuspended in lysis buffer containing complete, EDTA-free protease inhibitor cocktail (Roche), incubated on ice for 20 min and homogenized by 4 min of sonication (50 cycles/50% intensity). DNAse treatment was not deployed. Amylose-bound fusion protein was eluted during batch purification according to the manufacturer’s protocol.

### Generation of antisera

GST-tagged recombinant PfPKRP protein was prepared from bacterial inclusion bodies as described previously [36], while GST-tagged recombinant PfCLK-4 and MaBP-tagged recombinant SR proteins were purified via chromatography as described above. Specific immune sera against the recombinant fragments of the respective kinases and the SR-proteins were generated by the initial immunization of 6 weeks-old female NMRI mice (Charles River Laboratories) with 100 µg recombinant protein emulsified in Freund’s incomplete adjuvant (Sigma-Aldrich) followed by a boost with 30 µg recombinant protein 4 weeks after immunization. Mice were anesthetized by intraperitoneal injection of a mixture of ketamine and xylazine according to the manufacturer’s protocol (Sigma-Aldrich), and immune sera were collected 10 days after the second immunization (boost) via heart puncture. Following sera collection the anesthetized mice were sacrificed via severing the cervical spine. The immune sera of three mice immunized with the same antigen were pooled; sera of three non-immunized mice were used as negative control. The antisera recognized the cognate recombinant protein (data not shown). Experiments for the generation of antisera in mice were approved by the animal welfare committees of the government of Lower Franconia, Germany (ref. no. 55.2-2531.01-58/09), and of the District Council of Cologne, Germany (ref. no. 84-02.05.30.12.097 TVA). The generation of mouse anti-PfCLK-1 and anti-PfCLK-2 antisera was described previously [11]. As a second source of PfCLK1-specific antibody, sera directed against the peptide sequence NRTKTSDTEDKKER (AA508-521) upstream of the catalytic domain were produced by immunization of two rabbits (Biogenes, Berlin). Specific PfCLK-3 rat antibody was raised by immunizing rats with peptide YKSKHEENSPDGDSY (AA30-44) and purified by protein G as described [15].

### Indirect immunofluorescence assay

Parasite preparations for indirect immunofluorescence assays (IFAs) included mixed asexual blood stages of *P. falciparum* F12 strain or mature gametocytes of NF54 strain. Preparations were air-dried on slides and fixed for 10 min either in −80°C methanol or, to label the SR proteins, with 4% paraformaldehyde (pH 7.4). For membrane permeabilization and blocking of non-specific binding, methanol-fixed cells were incubated in 0.01% saponin, 0.5% bovine serum albumin faction V (BSA) and 1% neutral goat serum (Sigma-Aldrich) in PBS for 30 min. Paraformaldehyde-fixed samples were permeabilized with 0.1% vol. Triton X-100 and 125 mM glycine (Carl Roth) in PBS for 30 min, followed by blocking with 3% BSA in PBS for 1 h. Preparations were then incubated for 2 h at 37°C with rat antisera against PfCLK-3 or mouse antisera against PfCLK-4 and the SR proteins. Antisera dilutions of 1∶50 to 1∶100 were used. Binding of primary antibody was visualized using fluorophore-conjugated goat anti-rat or anti-mouse antibodies (Alexa Fluor 488; Molecular Probes). Asexual blood stage parasites were highlighted with rabbit immune sera specific for the merozoite surface protein PfMSP-1 (ATCC), while gametocytes were highlighted with rabbit anti-Pfs230 antisera, followed by incubation with fluorophore-conjugated goat anti-rabbit antibodies (Alexa Fluor 594; Molecular Probes). In the co-localization experiments, PfCLK-1 was immunolabelled in the young schizont stages using the respective rabbit antisera (dilution of 1∶100) in combination with Alexa Fluor 594 goat anti-rabbit antibody. Nuclei were highlighted by incubating the specimens with Hoechst nuclear stain 33342 (Molecular Probes) for 1 min. Labelled specimens were examined by confocal laser scanning microscopy using a LEICA TCS SP5 or an Olympus BX41 fluorescence microscope in combination with a ProgRes Speed XT5 camera. Digital images were processed using Adobe Photoshop CS software. Quantifications of schizonts (highlighted by anti-MSP-1 antisera or Hoechst nuclear stain) or gametocytes (highlighted by anti-Pfs230 antisera) positive for the PfCLKs or the SR proteins were performed in triplicate (40–60 parasites counted per well).

### Western blot analysis

Mixed blood stage parasites of *P. falciparum* strain NF54 were harvested and treated with 0.15% saponin for erythrocyte lysis. Parasite nuclear pellet and cytoplasmic fractions were prepared as described previously [11]. Parasite pellets, the nuclear pellet and cytoplasmic fractions as well as pellets of non-infected erythrocytes or immunoprecipitated bead-bound proteins (see below) were washed with PBS, resuspended and sonicated in lysis buffer (20 mM Tris-HCl pH 8.0, 10 mM EDTA pH 8.0, 400 mM NaCl, 1 mM PSMF, 10 mM β-glycerophosphate, 10 mM NaF, 0.25% Triton X-100) supplemented with a protease inhibitor cocktail (Roche Diagnostics). Parasite proteins were separated by SDS-PAGE electrophoresis and transferred to Hybond ECL nitrocellulose membrane (Amersham Biosciences) according to the manufacturer’s protocol. Membranes were blocked for non-specific binding by incubation in Tris-buffered saline containing 5% skim milk and 1% BSA, followed by immune recognition for 2 h at RT with rabbit anti-PfCLK-1, rat anti-PfCLK-3 or mouse anti-PfCLK-4, anti-Pf39 [36] or anti-Pfalpha-5 antisera [37]. For control sera from non-immunized animals were used. After washing, membranes were incubated for 1 h at RT with an alkaline phosphatase-conjugated secondary antibody (Sigma-Aldrich) and developed in a solution of nitroblue tetrazolium chloride (NBT) and 5-bromo-4-chloro-3-indoxyl phosphate (BCIP; Sigma-Aldrich) for 5–30 min. Scanned blots were processed using Adobe Photoshop CS software.

### Immunoprecipitation assay

Mixed *P. falciparum* NF54 asexual blood stage parasites were harvested and treated with 0.15% saponin for erythrocyte lysis. Parasite pellets were resuspended in 150 µl lysis buffer and sonicated with 50% amplitude and 50 cycles followed by centrifugation at 13,000×g at 4°C for 10 min. Pre-clearing of lysates was carried out by consecutive incubation with 5% vol. serum of non-immunized mouse, rabbit or rat, and 20 µl of protein G-beads (Santa Cruz Biotechnology) for 30 min each at 4°C. Lysates were then incubated for 2 h with the respective kinase-specific antisera (see above) or anti-Pf39 antisera under constant agitation at 4°C. Subsequently the antibody-antigen complexes were incubated over night with 20 µl of protein G-beads at 4°C. The beads were centrifuged, washed four times with 1×PBS and were subsequently deployed in Western blotting (see above) or the kinase activity assay (see below). Gels were stained after resolving with Coomassie Blue Stain and scanned for digital processing using Adobe Photoshop CS software.

### Kinase activity assay

Kinase reactions of 30 µl were carried out in a standard kinase buffer (20 mM Tris-HCl, pH 7.5, 20 mM MgCl_2_, 2 mM MnCl_2_, 10 mM NaF, 10 mM β-glycerophosphate, 10 µM ATP and 0.1 MBq [γ-^32^P]-ATP), using immunoprecipitated endogenous kinases as well as 5–10 µg of the respective substrate. When the PfPKRP-specific immunoprecipitates were used in the assays, 50 mM of bovine calmodulin (CaM; dissolved in water; CalBiochem) were added to the reaction. Recombinantly expressed His_6_-tagged protein kinase 6 (rPK6), purified as previously reported [38], was used as a positive control for the kinase activity assay utilizing exogenous substrates. For testing of the CLK inhibitors, sorbitol-synchronized ring stages were incubated with the inhibitors at IC_50_ concentrations for 12 h before immunoprecipitation. In the assays, exogenous substrates (histone H1, myelin basic protein (MBP), and α- and β-casein; Sigma-Aldrich), and the recombinant SR proteins used as phosphoacceptor substrates were added to the reaction. The recombinant SR proteins were purified via chromatography as described above. For negative control, purified MaBP-tag alone was used for substrate in the kinase activity assays. An additional negative control, in which the parasite lysate was replaced by the same volume of PBS (PBS control), was used to exclude unspecific phosphorylation of reaction components. Reactions were incubated at 37°C for 1 h under constant agitation and terminated by addition of reducing Laemmli buffer for 10 min at 95°C. Samples were separated on 12% SDS-PAGE, dried by means of vacuum gel drying and exposed to X-ray films for 48–90 h at −20°C. For quantification of inhibition of phosphorylation, the mean grey values (MGV) of the phosphorylation bands were measured using the ImageJ program. Background values were subtracted and the MGVs for the DMSO control were set to 100% to calculate the relative MGV (rMGV).

### Malstat assay

The CLK inhibitors were screened for antiplasmodial activity against *P. falciparum* strain 3D7 at concentrations of 6.4 nM–500 µM, using the Malstat assay as described [39]–[41]. Sorbitol-synchronized ring stages were plated in triplicate in 96-well plates (200 µl/well) at a parasitemia of 1% in the presence of the compounds. Chloroquine (diphosphate salt, Sigma-Aldrich; dissolved in double-distilled water) served as positive control in all experiments. Incubation of parasites with DMSO alone at a concentration of 0.5% vol. was used as negative control. Treated parasites were cultivated *in vitro* for 72 h, resuspended and aliquots of 20 µl were transferred to a new plate. 100 µl of the Malstat reagent was added to initiate the conversion of lactate to pyruvate by parasite lactate dehydrogenase (pLDH) in the presence of the co-factor APAD. The reduced APAD (APADH) formed during the reaction is used for the assessment of pLDH activity by adding a 20 µl of a mixture of NBT (nitro blue tetrazolium)/Diaphorase (1∶1; 1 mg/ml stock each) to the Malstat reaction. The APADH and the NBT form a purple coloration and the absorbance was measured at OD (630 nm). Each compound was tested 2 to 4 times, and the IC_50_ values were calculated from variable-slope sigmoidal dose-response curves using the GraphPad Prism program version 5.

### Stage of inhibition assay

The compounds CHX, KH-CARB-10, KH-CARB-11, KH-CARB-13xHCl, and gea-27 were added to synchronized ring stage parasites (T0) in IC_50_ (0.8, 7.5, 6.1, and 4.0 µM, respectively) and IC_80_ (4.0, 37.0, 30.0, and 20.0 µM respectively) concentrations, and blood smear samples were taken at 24, 36, 48, and 60 h of incubation with CHX and at 24 h of incubation in case of the other inhibitors. The numbers of ring stages, trophozoites and schizonts as well as of dead parasites were counted for a total number of 100 infected erythrocytes for each setting.

### Gametocyte toxicity assay

*P. falciparum* NF54 parasites were grown at high parasitemia to favour gametocyte formation. Upon appearance of stage II gametocytes, 1 ml of culture was aliquoted in triplicate in a 24-well plate in the presence of compounds at the respective IC_50_ concentrations. The gametocytes were cultivated for 7 d and the medium was replaced daily. For the first 48 h of cultivation, the gametocytes were treated with CLK inhibitors; subsequently the medium was compound-free. At day 7, Giemsa-stained blood smears were prepared and the gametocytemia was evaluated by counting the numbers of gametocyte stages IV and V in a total number of 1000 erythrocytes. Negative controls were performed with 0.5% vol. of DMSO. Chloroquine was used as additional negative control, while epoxomicin was used as positive control [41]. Two independent experiments in triplicates were conducted and the mean gametocytemia was calculated for each compound. Data from the experimental cultures was normalized to the DMSO control, which was set to 100%, and the student’s t-test was performed for statistical analysis using the Microsoft Excel 2010 program.

### Exflagellation inhibition assay

A volume of 100 µl of mature NF54 gametocyte cultures was pre-incubated with CLK inhibitors in concentrations ranging between 0.1 µM-1 mM for 15 min at 37°C. Each sample was then transferred to RT and 100 µM of xanthurenic acid was added for activation. After another 15 min, the numbers of exflagellation centers were counted in 30 optical fields using a Leica DMLS microscope by 400-fold magnification. Two independent experiments were performed in duplicates and the inhibition of exflagellation was calculated as a percentage of the number of exflagellation centers in compound-treated cultures in relation to the number of exflagellation centers in untreated controls with 0.5% vol. of DMSO. The IC_50_ values were calculated from variable-slope sigmoidal dose-response curves using the GraphPad Prism program version 4.

## Results

### The PfCLKs show homologies with yeast SR protein kinase Sky1p

The genome of *P. falciparum* encodes four proteins clustering within the CLK family, termed PfCLK-1-4 [9]. In PfCLK-1-3, the predicted kinase catalytic domains are located at the C-terminus; the catalytic domain of PfCLK-4, on the other hand, starts at the N-terminus and spans more than half of the protein (see Fig. 1A). While *in silico* analysis of gene sequences previously revealed two nuclear localization signals for PfCLK-1 and one for PfCLK-2 upstream of the C-terminal catalytic domains [11], PfCLK-3 and PfCLK-4 do not comprise such signals.

The catalytic domain sequences of the four PfCLKs showed homologies with the *Saccharomyces cerevisae* SR protein kinase Sky1p [42], as was shown by sequence alignment (Fig. S1) [11]. The sequence DLKPxN with the conserved Aspartate 126 is present and is considered to be the catalytic base. The loop at positions 169–193 signifies the activation segment, starting with Aspartate 169 and ending with sequence APE in PfCLK-1, PfCLK-3, PfCLK-4 and SPE in PfCLK-2 and Sky1p. The ATP-binding domain GXGXXG is present at positions 8–13 for PfCLK-1 and GXGXXS for PfCLK-3, PfCLK-4 and Sky1p, but is missing in PfCLK-2. Furthermore, sequence alignment with Sky1p revealed matches between substrate binding residues of the kinases with the substrate binding site of Sky1p, including Arginine 187, Tyrosine 189, Arginine 190 and Glutamate 215 (Fig. S1).

### The PfCLKs and the SR proteins are expressed in the blood stages of *P. falciparum*

**Figure 3:**
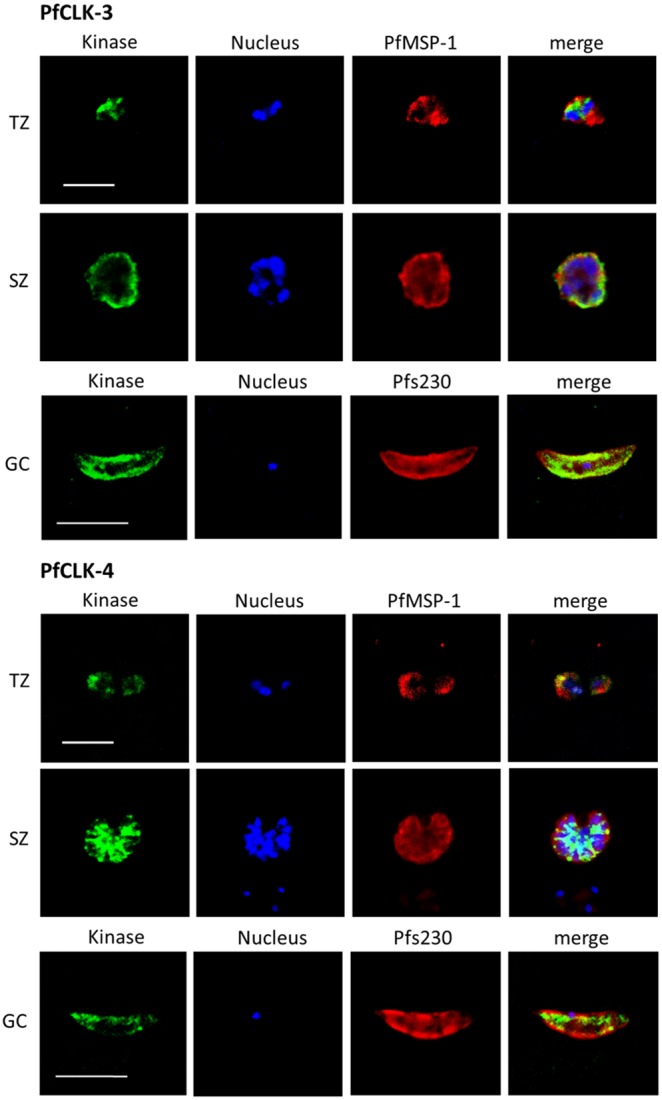
Subcellular localization of PfCLK-3 and PfCLK-4 in the blood and gametocyte stages. Mixed asexual blood stage cultures containing trophozoites (TZ) and schizonts (SZ) and mature gametocyte (GC) cultures were fixed with methanol and prepared for IFA, using rat antisera against PfCLK-3 and mouse antisera against PfCLK-4 (green). The parasite nuclei were highlighted by Hoechst staining (blue). The asexual blood stages were labelled with rabbit antisera against PfMSP-1 and gametocytes with rabbit antisera against Pfs230 (red). Bar, 5 µm.

Previous studies showed that PfCLK-1 and PfCLK-2 are present in the asexual blood stages and in gametocytes of *P. falciparum*. Here, PfCLK-1 can be found in the nucleus of trophozoites, while in schizonts and gametocytes the kinase is present in nucleus and cytoplasm. PfCLK-2 is detected in the nucleus and the cytoplasm of the asexual blood and gametocyte stages [11]. Via IFA we now show that PfCLK-3 and PfCLK-4 are mainly present in the nucleus of trophozoites, while in schizonts and gametocytes both kinases are primarily located in the cytoplasm (Fig. 3). Particularly for PfCLK-3 a rim-associated labelling pattern was observed in the latter stages. In the IFAs, the asexual blood stage parasites and the gametocytes were highlighted by labelling of plasmalemma-associated proteins, i.e. PfMSP-1 and Pfs230, respectively.

**Figure 4:**
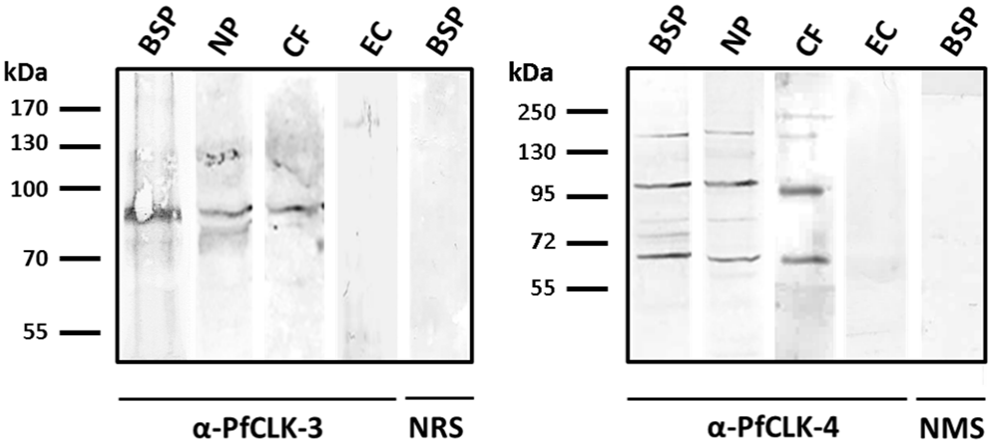
Expression of PfCLK-3 and PfCLK-4 in the cytoplasmic and nuclear fractions of blood stage parasites. Western-blot analyses on lysates of mixed blood stage parasites (BSP) as well as of nuclear pellet (NP) or cytoplasmic fraction (CF) of BSP using rat antisera against PfCLK-3 or mouse antisera against PfCLK-4 detected the full length protein bands for PfCLK-3 and PfCLK-4 of approximately 80 and 150 kDa, respectively. For PfCLK-4, two additional protein bands of approximately 100 and 70 kDa are present. No proteins were detected by the anti-PfCLK antisera in lysates of non-infected erythrocytes (EC). Immunoblotting with sera from non-immunized rat (NRS) or mouse (NMS) did not result in the labelling of any protein bands.

The presence of PfCLK-3 and PfCLK-4 in the nucleus and cytoplasm of blood stage parasites was subsequently confirmed by Western blot analysis. Immunoblotting of blood stage parasite lysate with rat anti-PfCLK-3 antisera labelled the full length kinase of approximately 80 kDa (Fig. 4). Full length PfCLK-3 was further detected, when nuclear pellet and cytoplasmic fractions were immunoblotted with the respective antibody. Immunoblotting of the blood stage lysate as well as nuclear pellet and cytoplasmic fractions with mouse anti-PfCLK-4 antisera, on the other hand, resulted in the labelling of three bands, the full-length kinase band of approximately 160 kDa and two additional bands with molecular weights of approximately 100 and 70 kDa, which might represent processing products (Fig. 4). Lysate of non-infected erythrocytes were used for control, and no protein bands were detected after immunoblotting with the respective anti-PfCLK antisera. Similarly, no protein bands were detected, when the blood stage parasite lysates were immunoblotted with sera of non-immunized animals (Fig. 4).

**Figure 5:**
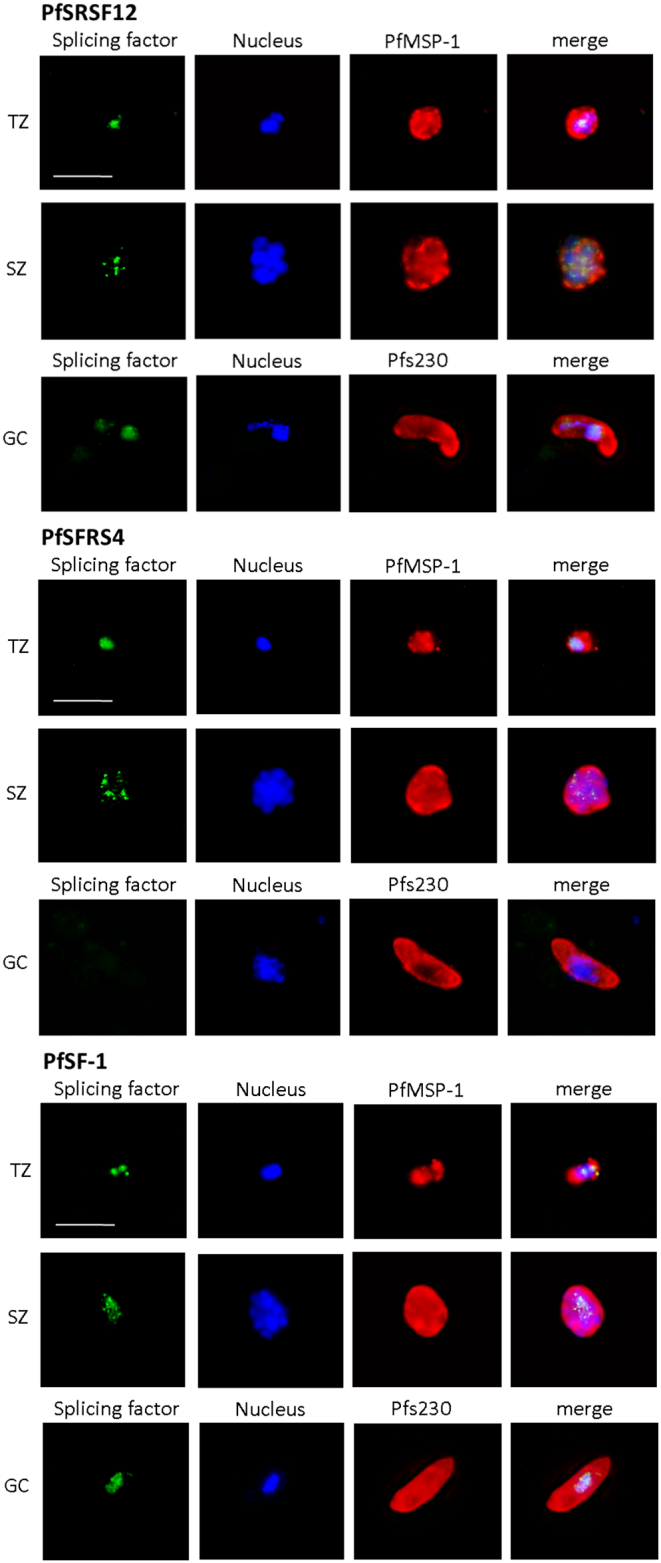
Subcellular localization of the SR proteins in the blood and gametocyte stages. Mixed asexual blood stage cultures containing trophozoites (TZ) and schizonts (SZ) and mature gametocyte (GC) cultures were fixed with paraformaldehyde and prepared for IFA, using mouse antisera against PfSRSF12, PfSFRS4 and PfSF-1 (green). The parasite nuclei were highlighted by Hoechst staining (blue). The asexual blood stages were labelled with rabbit antisera against PfMSP-1 and gametocytes with rabbit antisera against Pfs230 (red). Bar, 5 µm.

**Figure 6:**
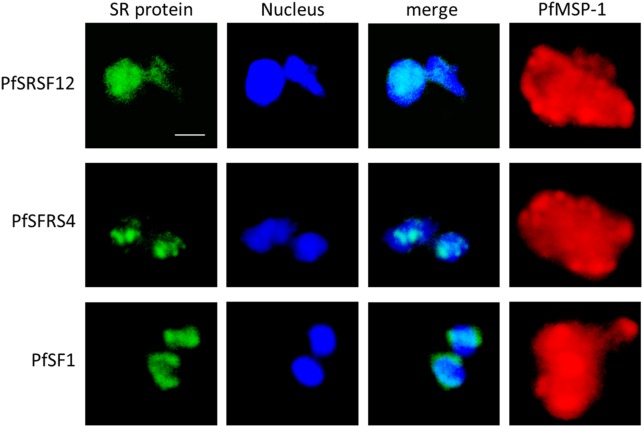
Localization of the SR proteins in the parasite nucleus. Transforming trophozoites (2-nuclei stages) were prepared for IFA as described in Fig. 5 and the localization of the SR proteins was investigated in the Hoechst-positive parasite nuclei. Bar, 5 µm.

We then investigated the blood stage-specific localization of three previously identified SR proteins, i.e. PfSRSF12, PfSFRS4 and PfSF-1 (see Fig. 1B) [14]. Transcriptome data available at PlasmoDB point to a predominant transcript expression in the trophozoite stage for all three SR proteins [17]. In accord with these data, IFAs, using respective antisera raised in mice, detected the three SR proteins in the trophozoite nucleus (Fig. 5, top rows). An additional minor labelling was observed in the nuclei of the schizont stages (Fig. 5, center rows). Furthermore, PfSRSF12 and PfSF-1 were present in the nucleus of gametocytes, while PfSFRS4 was not detected in these stages (Fig. 5, bottom rows). In depth analysis of the localization of the SR proteins in transforming trophozoites (2-nuclei stage) confirmed that the splicing factors are present in distinct areas of the parasite nuclei, while they cannot be detected in the cytoplasm of the parasites (Fig. 6).

We subsequently performed co-localization experiments between the three SR proteins, using the respective mouse antisera, and PfCLK-1, using antisera raised in rabbit. The co-localization experiments confirmed that the SR proteins are solely present in the nuclei, while in the early schizont stage, PfCLK-1 is present both in the parasite nuclei and the cytoplasm. Co-localization of PfCLK-1 with the three SR proteins can be detected in distinct nuclear regions (Fig. S2A).

In a subsequent step, the numbers of parasites positive for the PfCLKs and the SR proteins were determined. When blood stage schizonts were highlighted by immunolabelling with anti-MSP-1 antibody or by Hoechst nuclear staining, 99±1.0% of schizonts labelled for PfCLK-1-3 or PfSF-1, 96±2% of schizonts labelled for PfCLK-4, 92±2.8% of schizonts labelled for PfSFRS4, and 94±0.6% of schizonts labelled for PfSRSF12. Further 100±0.9% of Pfs230-positive gametocytes labelled for PfCLK-1, 98±1.1% of gametocytes labelled for PfCLK-2, 84±2.3% of gametocytes labelled for PfCLK-3, 94±1.1% of gametocytes labelled for PfCLK-4, 96±1.6% of gametocytes labelled for PfSF-1, and 96±0.6% of gametocytes labelled for PfSRSF12.

No labelling was detected, when serum of non-immunized mice was used in the IFAs. Further, IFAs using mouse antisera directed against the GST- and MaBP-tags did not result in any labelling of the blood stage parasites (Fig. S2B).

### The PfCLKs mediate phosphorylation of SR proteins

We aimed to determine the phosphorylation activity of precipitated PfCLKs obtained from asexual blood stage cultures. As described previously, immunoblotting of blood stage parasite lysate with mouse or rabbit anti-PfCLK-1 antisera resulted in a processed kinase band of approximately 60 kDa (Fig. S3A), while mouse anti-PfCLK-2 antisera detected a full-length protein band at 150 kDa [11]. Furthermore, immunoblotting of blood stage parasite lysate with rat anti-PfCLK-3 or mouse anti PfCLK-4 antisera detected full-length kinase bands of approximately 80 and 160 kDa, respectively (see above, Fig. 4). When the PfCLKs were immunoprecipitated from the blood stage parasite lysate using the respective antisera, the full-length kinase bands for PfCLK-2-4 were detected, when the same antisera were used for immunoblotting (Fig. S3B). It was not possible, however, to detect the processed 60 kDa band typical for PfCLK-1, since protein bands of this molecular weight interfere with the protein band of the heavy chain of the precipitating antibody, which is running as a smear at a molecular weight of approximately 55 kDa (Fig. S3B). For control, sera of non-immunized animals were used in immunoblotting and no precipitating protein bands (with the exception of the heavy chain antibody band) were detected (Fig. S3C). Similarly, no precipitated protein bands were detected, when mouse antisera directed against the endoplasmic reticulum-associated protein Pf39 [43] or the proteasome subunit alpha 5 (Pfalpha-5) [37] were used for immunoblotting (data not shown).

**Figure 7:**
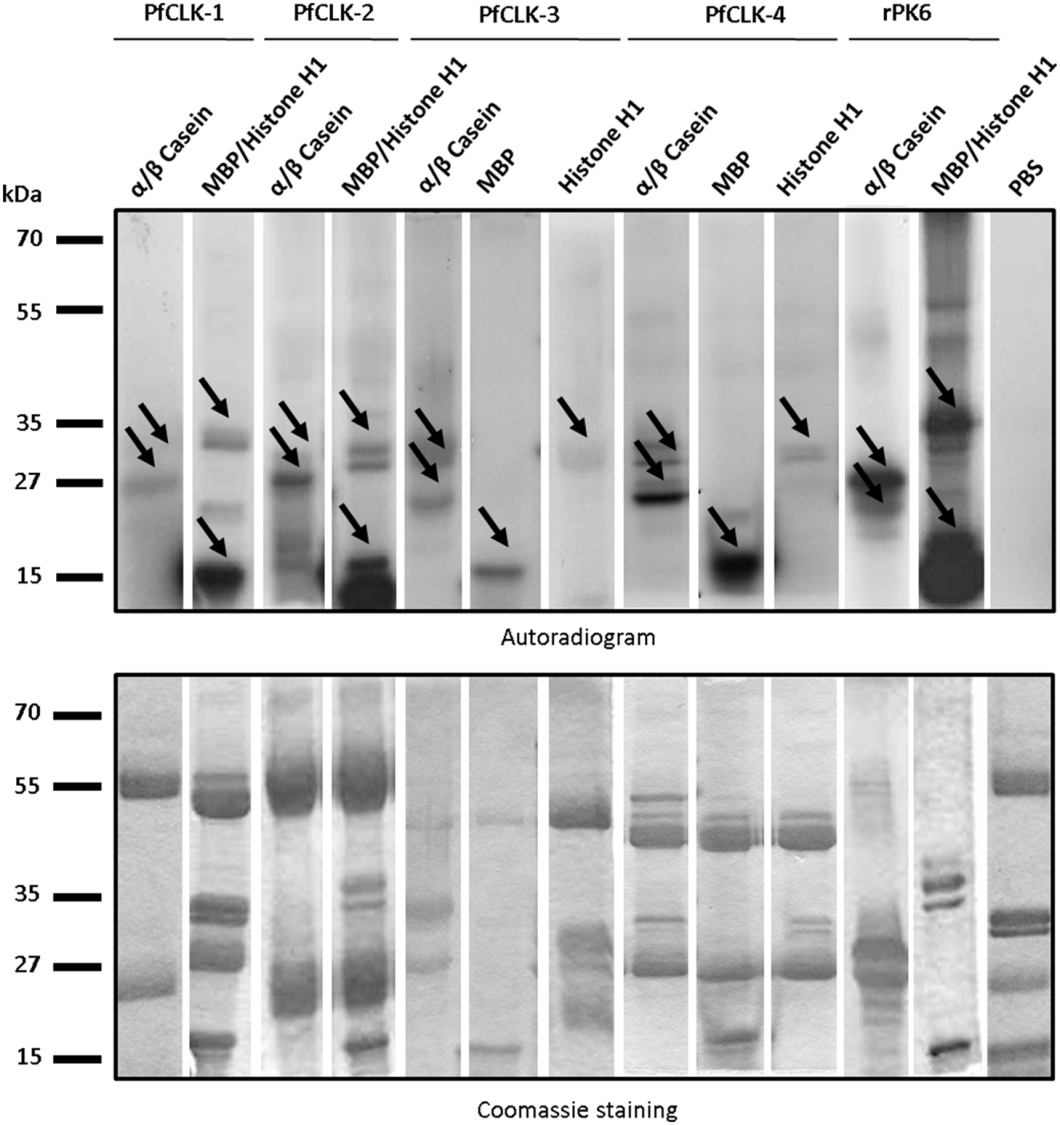
Phosphorylation of exogenous substrates by immunoprecipitated PfCLKs. Kinase activity assays were deployed to detect phosphorylation of the substrates histone H1, MBP and α/β casein (∼33, 18, and 28/34 kDa, respectively; indicated by arrows) by the four immunoprecipitated PfCLKs (autoradiogram, upper panel), using the PfCLK-specific respective mouse, rabbit or rat antisera. Assays without precipitated proteins (PBS control) were used for negative controls. Recombinant protein kinase 6 (rPK6) was used for positive control. Coomassie blue staining (lower panel) of radiolabelled SDS-gels was used as a loading control.

The immunoprecipitated PfCLK proteins were used in the kinase activity assays as described previously [11], adding as exogenous substrates histone H1, MBP, or α/β casein, as well as radiolabelled [γ-^32^P] ATP. All PfCLKs were associated with phosphorylation activity of all three substrates (Fig. 7, upper panel). Recombinant PfPK6 was used as a positive control [38] and also exhibited strong phosphorylation activity of the three exogenous substrates. As negative control, the assay was performed without precipitated kinases (PBS control). Coomassie blue staining of radiolabelled gels were used for loading control (Fig. 7, lower panel).

**Figure 8:**
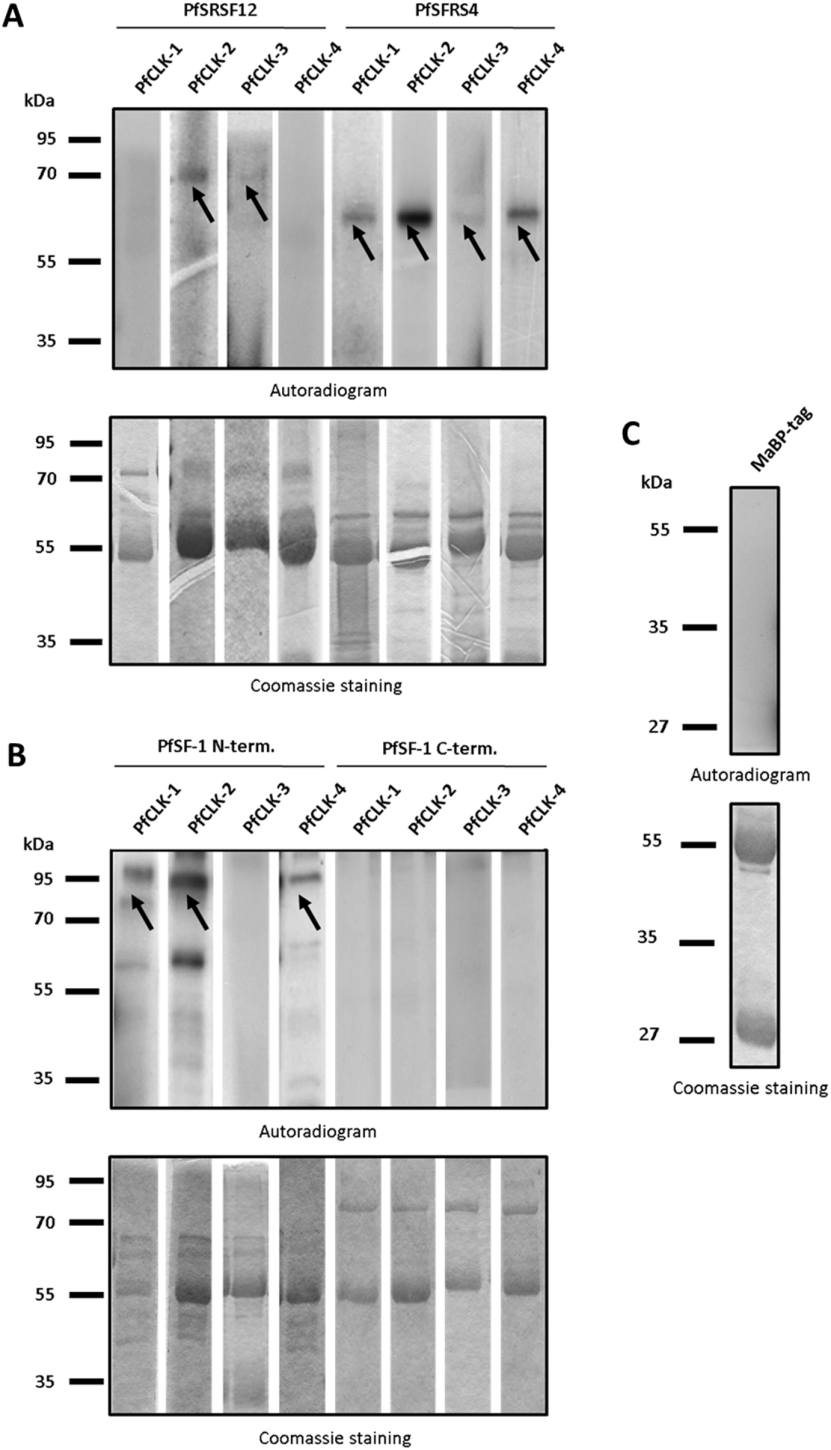
Phosphorylation of plasmodial SR proteins by immunoprecipitated PfCLKs. A. Kinase activity assays were deployed to detect phosphorylation of recombinant PfSRSF12 and PfSFRS4 (∼73 and 65 kDa, respectively; indicated by arrows) by two or more of the PfCLKs (autoradiogram; upper panel). B. The N-terminal part (∼95 kDa; indicated by arrows), but not the C-terminal part (86 kDa) of recombinant PfSF-1 was phosphorylated by immunoprecipitated PfCLKs. An additional phosphorylation signal of truncated N-terminal PfSF-1 was visible at approximately 60 kDa. C. MaBP-tag alone (43 kDa) as substrate was used as negative control. Shown here is an assay using PfCLK-3-specific immunoprecipitate, similar results were obtained with immunoprecipitates of other PfCLKs (not shown). Coomassie blue staining (lower panels) of radiolabelled SDS gels was used as a loading control.

Subsequently we wanted to determine if the plasmodial SR proteins PfSRSF12, PfSFRS4, and PfSF-1 function as *in vitro* substrates for the immunoprecipitated PfCLKs. Peptides comprising the RS-rich domains were bacterially expressed as tagged fusion proteins. For PfSF-1, two recombinant proteins were expressed, comprising either the N-terminal part with the RS-rich domain or the C-terminal part, which includes the RNA recognition motif (see Fig. 1B). The SR proteins were purified via affinity chromatography before deploying these as substrates in the above described kinase activity assays. The assays revealed that recombinant PfSRSF12 was phosphorylated by anti-PfCLK-2 and anti-PfCLK-3 precipitate (Fig. 8A, upper panel), whilst recombinant PfSFRS4 was phosphorylated by all PfCLK-specific precipitates. When the immunoprecipitates were incubated with the N-terminal fraction of PfSF-1, phosphorylation signals were detected with the exception of the PfCLK-3 precipitate. In contrast, no phosphorylation signals were detected, when the immunoprecipitates were incubated with the C-terminal part of PfSF-1, which did not comprise the RS-rich motif (Fig. 8B, upper panel). Purified MaBP-tag was used as a substrate instead of the recombinant SR proteins and served as a negative control in the assays, and no phosphorylation of MaBP was observed (Fig. 8C, upper panel). Coomassie blue staining of radiolabelled gels was used as a loading control (Fig. 8, lower panels).

### CLK inhibitors impair the phosphorylation activity of the PfCLKs

Previous attempts to knock-out the *pfclk* genes via single cross-over gene disruption in order to study the function of the PfCLKs was unsuccessful [11], [15]. However, locus modification (tagging each sequence with a Myc epitope to the 3′-end) was achieved [11], [15] (S. Kern, G. Pradel, unpublished observations), demonstrating that the genomic loci are accessible for recombination. These data lead to the conclusion that the PfCLKs have essential roles for the parasite blood stages, prohibiting the survival of PfCLK knock-out mutants.

In order to functionally analyse the role of the PfCLKs for the *P. falciparum* blood and sexual stages, we aimed at a chemical inhibition of the kinases. We screened a small compound library of 63 human CLK inhibitors [22], [44] for antiplasmodial activity, using the Malstat assay (Table S2). We identified five compounds, i.e. the oxo-β-carbolines KH-CARB-10, KH-CARB-11, KH-CARB-13xHCl, and the aminopyrimidines C-117 and gea-27, which exhibited half-maximal growth inhibitory concentrations (IC_50_) in the low micromolar range (Table 1). We further screened the antiseptic chlorhexidine (CHX) for antiplasmodial activities, because it has previously been reported to act on *P. falciparum* [45] as well as on CLKs [46], [47]. In the Malstat assay, CHX exhibited an IC_50_ value in the nanomolar range (Table 1).

**Table 1:** Antiplasmodial and transmission blocking activities of the CLK inhibitors.

Compound	Class	IC_50_ (Malstat) [µM]	IC_50_ (EIA) [µM]
KH-CARB10	oxo-β-carboline	7.2±2.40	14.0±3.72
KH-CARB11	oxo-β-carboline	6.1±1.09	15.8±0.84
KH-CARB13xHCl	oxo-β-carboline	4.4±1.84	13.8±6.22
C-117	aminopyrimidine	7.5±2.76	67.5±13.03
gea-27	aminopyrimidine	5.2±0.35	154.2±56.43
CHX	antiseptic	0.6±0.20	19.8±0.93
Chloroquine	4-aminoquinoline	0.04±0.013	N/A

**Figure 9:**
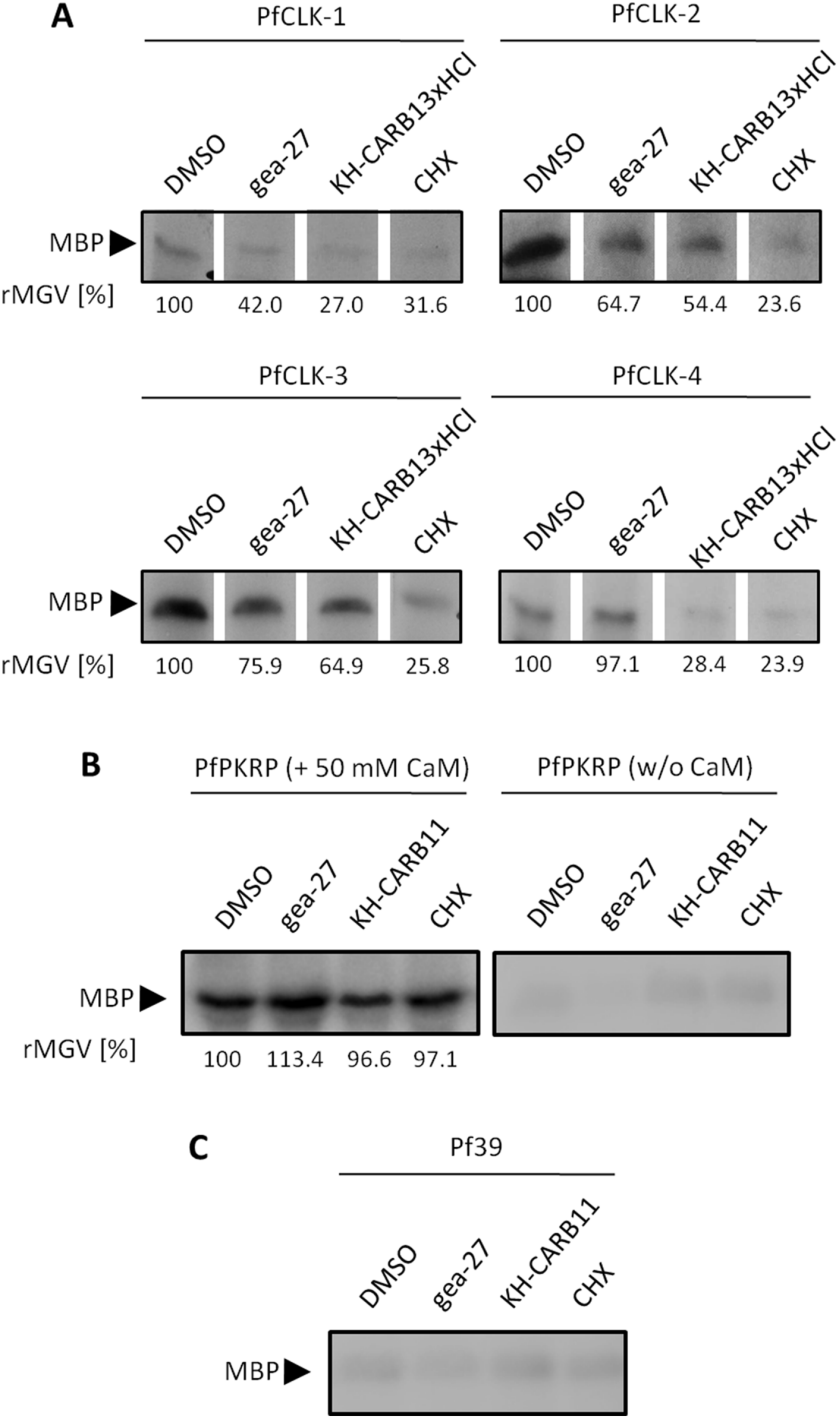
Effect of CLK inhibitors on MBP phosphorylation. A. Kinase activity assays were deployed to detect MBP phosphorylation by inhibitor-treated immunoprecipitated PfCLKs. The parasites were incubated with the CLK inhibitors at IC_50_ concentrations or 0.5% vol. DMSO for 12 h prior to the assays. The phosphorylation signals were measured as rMGV (MGV of DMSO set to 100%). B. PfPKRP-specific immunoprecipitate phosphorylates MBP in the presence but not absence of 50 mM CaM, independent from prior incubation of the parasites with the CLK inhibitors. C. Pf39-specific immunoprecipitate was used as a negative control in the assays.

In order to confirm that the inhibitors act on the PfCLKs, we tested these for their inhibitory effect on PfCLK-mediated phosphorylation. Blood stage parasites were incubated with the three inhibitors gea-27, KH-CARB-13xHCl, and CHX at IC_50_ concentrations for 12 h. DMSO was used for negative control. The kinase activity assays were conducted as described above, using MBP for substrate. The signals for phosphorylated MBP were detected by autoradiography and the rMGV of the phosphorylation signals was measured. The assays revealed a reduction in the rMGV by 24.1–76.4%, when the parasites were incubated with the inhibitors prior to the assay (Fig. 9A). For gea-27, no effect on the phosphorylation activity of PfCLK-4 was detected.

As control, the assays were carried out on immunoprecipitates using antisera against the CaM-dependent protein kinase-related protein PfPKRP [15]. The 295 kDa PfPKRP has an N-terminal catalytic domain (Fig. S4A) and is a homolog of the *P. berghei* PKRP, which plays a role for parasite transmission to the mosquito [48]. Because protein expression of PfPKRP has not yet been described in the *P. falciparum* blood stages, we tested the antisera in IFA prior to use in the kinase activity assays. PfPKRP was detected in the asexual blood stage schizonts (Fig. S4B). In gametocytes, the kinase is present throughout maturation from stage II to stage V, and here is localized in the cytoplasm (Fig. S4B, C). Because PfPKRP was annotated as a CaM-dependent kinase, the experiments were conducted with and without addition of 50 mM CaM. In the presence of CaM, the PfPKRP-specific immunoprecipitate was able to phosphorylate MBP (Fig. 9B). In reactions lacking CaM, no specific phosphorylation of MBP was detected, indicating that the activity of PfPKRP is CaM-dependent. Noteworthy, no differences in the MBP phosphorylation signals were detected between precipitates of parasites treated with the CLK inhibitors and DMSO-treated control parasites (Fig. 9B). The PfPKRP control experiment on the one hand demonstrates that the CLK inhibitors do not inhibit PfPKRP, and on the other hand proves that the decrease in the phosphorylation signal of the PfCLK-specific precipitates seen after treatment with the CLK inhibitors is not caused by a general reduced viability of the inhibitor-treated parasites. As a second negative control, antisera against Pf39 [43] was used for immunoprecipitation. Pf39-specific precipitate did not phosphorylate MBP, showing that in the absence of immunobound kinases, the parasite precipitate has no phosphorylation activity (Fig. 9C).

### PfCLK inhibitors block schizogony and sexual stage development

**Figure 10:**
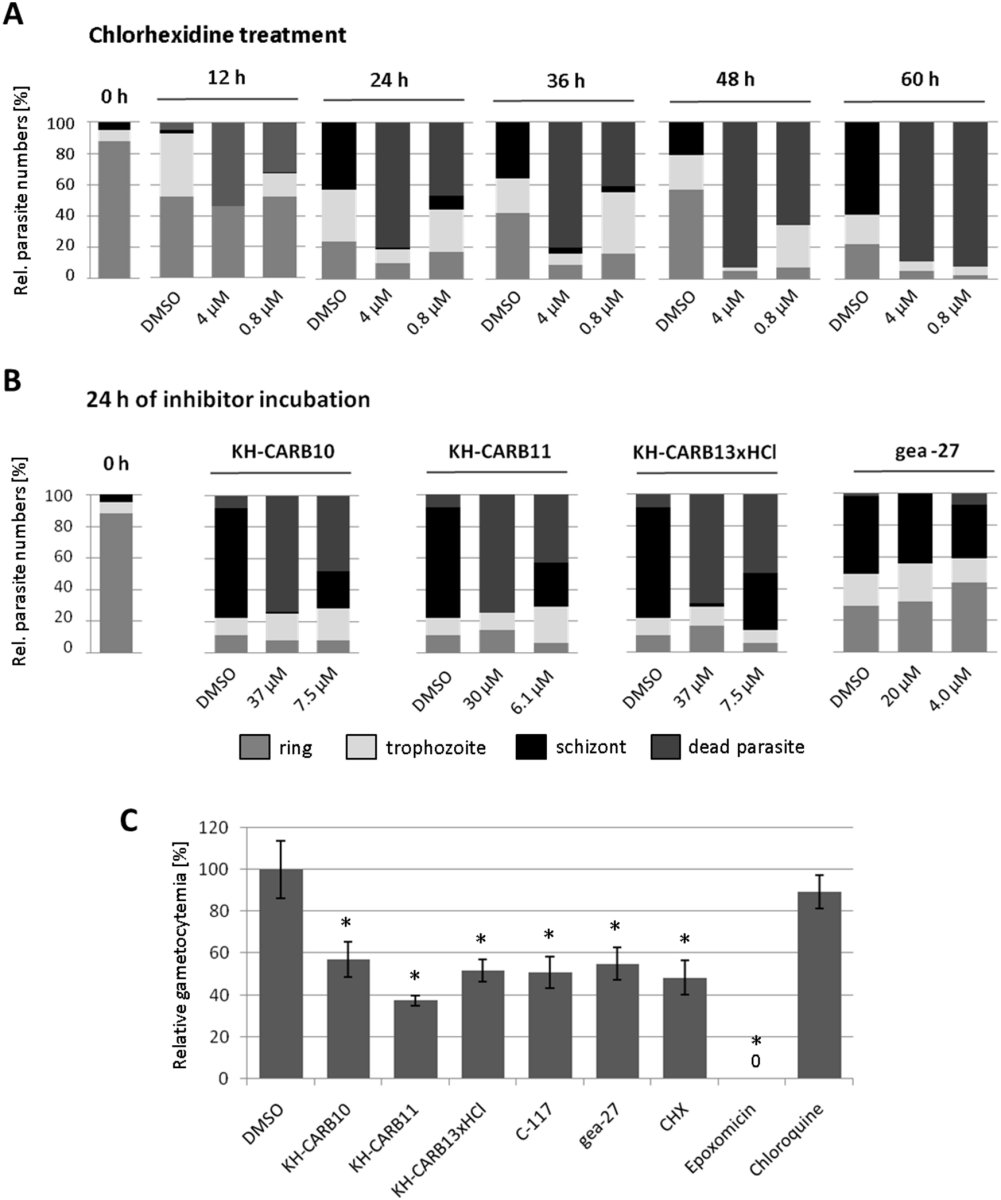
Effect of CLK inhibitors on blood stage parasites. A. Stage of growth inhibition of asexual blood stages between 12–60 h of CHX treatment. CHX at approximate IC_50_ and IC_80_ concentrations or 0.5% vol. of DMSO was added to synchronized parasites at the ring stage. Giemsa stained blood smears were prepared at six time points between 0–60 h of incubation with CHX and the numbers of ring stages, trophozoites, schizonts and dead parasites were counted. Histograms indicate the percentages of developmental stages present in the respective blood smears. B. Parasites were treated with CLK inhibitors as described above and the stage of growth inhibition was determined at 24 h of compound incubation. A total number of 100 parasites were counted in A and B for each condition. C. Compounds at IC_50_ concentrations or 0.5% vol. of DMSO were added to stage II gametocyte cultures for two days. After seven days, the numbers of stage IV and V gametocytes were counted in a total of 1000 erythrocytes and correlated to the gametocyte numbers of DMSO control (set to 100%). *, significant reduction of gametocyte numbers (p<0.001, student’s t-test). Epoxomicin was used as positive and chloroquine was used as a negative control.

After confirming the PfCLK-specific inhibitory activity, we aimed to determine at which developmental stage the parasites become arrested by the inhibitors. This was investigated using the stage-of-inhibition assay as previously described [41], [49]. The most active inhibitor, CHX, was given to the synchronized ring stage parasites (T0) at approximate IC_50_ and IC_80_ concentrations and Giemsa smears were taken at 24–60 h of CHX incubation. The Giemsa smears showed that the CHX-treated parasites developed to trophozoites but that the majority of the parasites died before entering the schizont stage. While a few parasites escaped the killing during the first replication cycle at the given concentrations, these died during trophozoites-to-schizont transformation of the second replication cycle (Fig. 10A). To investigate, if a similar killing mechanism applies to the other CLK inhibitors, we determined the stage of inhibition for KH-CARB-10, KH-CARB-11, KH-CARB-13xHCl, and gea-27 after 24 h of incubation of parasites with the respective compound at the approximate IC_50_ and IC_80_ concentrations. In all cases, the blood stage parasites died, once they entered schizogony (Fig. 10B). The lowest killing effect at 24 h of compound incubation was observed for gea-27, here schizonts were observed in parasite samples treated with IC_50_ and IC_80_ concentrations of the compound (Fig. 10B).

Once the antiplasmodial activity of the CLK inhibitors was demonstrated, we investigated their effect on the sexual transmission stages of *P. falciparum*. Firstly, the gametocytocidal activity of the compounds was tested, using the gametocyte toxicity test as previously described [41]. The CLK inhibitors significantly reduced the maturation of gametocytes by 40–60% compared to DMSO-treated controls. KH-CARB-11 showed the highest gametocytocidal activity with a reduction of mature gametocytes by 62.5% (Fig. 10C). Chloroquine was used as a negative control, while the proteasome inhibitor epoxomicin was used as a positive control. The CLK inhibitors further impaired exflagellation of *P. falciparum*, as demonstrated by exflagellation inhibition assay. The strongest inhibitory activities on male gametogenesis were shown for KH-CARB-10, KH-CARB-11, and KH-CARB-13xHCl with IC_50_ values between 10–20 µM (Table 1). The combined data indicate that the PfCLKs play essential roles for erythrocytic schizogony, gametocyte differentiation and gametogenesis.

## Discussion

CLKs are dual specificity protein kinases that phosphorylate SR proteins involved in pre-mRNA processing. These, when not in action, reside in the nuclear speckles, but shuttle between the nucleus and the cytoplasm during splicing. The activity of SR proteins is controlled by their phosphorylation status, and a change in phosphorylation changes the ability of SR proteins to interact with other proteins. Because the choice of splice sites during pre-mRNA processing is regulated by the concentration and the phosphorylation status of the SR proteins, CLKs play an indirect role in governing splice site selection emphasizing their importance for AS (reviewed in [50]).

The four PfCLKs of *P. falciparum* show homologies with yeast Sky1p, a kinase involved in mRNA splicing and mRNA transport [42], [51]. The kinases are abundantly expressed in the asexual blood stages of *P. falciparum*. In the trophozoites, the PfCLKs are present in the nucleus, where they co-localize with the plasmodial SR proteins PfSRSF12, PfSFRS4 and PfSF-1. During schizogony and gametocyte development, the kinases relocate from the nucleus to the parasite cytoplasm. Noteworthy, PfCLK-1 and PfCLK-2 possess nuclear localization signal sites and are predicted to be localized in the parasite nuclear speckles. A nucleus-cytoplasm passage was demonstrated for both kinases on the ultrastructural level [11]. The combined immunohistological data let us conclude that the PfCLKs accompany splicing factors during the shuttle between nucleus and cytoplasm or phosphorylate substrate in the cytoplasm in dependence on the developmental stage of the blood stage parasite.

In previous studies we were unable to generate *P. falciparum* parasites with disrupted *pfclk* loci, while the insertion of a tagging sequence was possible for all *pfclk* genes [11], [15] (S. Kern, G. Pradel, unpublished observation). The inability to knock out a kinase gene locus, together with the ability to modify the allele in a way that does not cause loss-of-function of the gene product, are strongly indicative of an essential role during the asexual blood cycle. The inability to disrupt a single *pfclk* gene further demonstrates that none of the PfCLKs can compensate for the loss of another PfCLK, indicating that the kinases have non-redundant functions. This is in accordance with the findings that multiple kinases are involved in SR protein phosphorylation in different cellular compartments. For example human ASF becomes phosphorylated by SRPK1 and SRPK2 in the cytosol, thus facilitating its nuclear import and accumulation in the nuclear speckles. There, a family of CLKs hyperphosphorylates the SR protein, mediating its release from the nuclear speckles and relocation to the region, in which splicing events occur [52]–[54]. Noteworthy, in the rodent malaria model *P. berghei*, the *pbclk4* gene (termed *srpk1* in this study) can be knocked out, resulting in impaired exflagellation [55]. In accordance with these findings we recently showed that PfCLK-4 is abundantly expressed in gametocytes, but down-regulated once gametogenesis has ceased [56], indicating that PfCLK-4 has a particular function during parasite transmission from the mammalian host to the mosquito vector.

We identified three plasmodial SR proteins, PfSRSF12, PfSFRS4, and PfSF-1, which function as substrates for the PfCLKs *in vitro*, and the four kinases appear to have different phosphorylation preferences for the three SR proteins. The three SR proteins are predominantly present in the nucleus of blood stage parasites, where they co-localize with the PfCLKs, and PfSRSF12 and PfSF1 can further be detected in gametocytes. Additionally, PfCLK-4 (SRPK1) was previously reported to phosphorylate AS factor SR1 [13]. For all of the plasmodial SR proteins, phosphorylation sites have been identified (Table S3) and the proteins are phosphorylated *in vivo*, as determined in the *P. falciparum* schizont stages [15], [57]–[59]. Noteworthy, a peptide of PfSF-1, which comprises the RNA recognition motifs, but not the RS-rich motifs, cannot be phosphorylated by any of the four PfCLKs, demonstrating the importance of the RS-rich motif for the interaction between SR protein and kinase.

Due to the inability to knock-out the *pfclk* genes by reverse genetics, we aimed to chemically knock-out the PfCLKs for functional studies. We identified five human CLK inhibitors as well as the antiseptic CHX as antiplasmodial agents and demonstrated their inhibitory effect on the PfCLKs via kinase activity assay. The inhibitors act on the *Plasmodium* parasites in the low micromolar range. While we demonstrate that the CLK inhibitors have no activity against the calmodulin-dependent kinase PfPKRP, other off-target effects of the inhibitors can currently not be excluded.

Morphological analyses on drug-treated parasites showed that the inhibitors arrest the parasites during the trophozoites-to-schizont transition. Furthermore, the inhibitors impaired gametocyte development and exflagellation. Particularly the KH-CARB inhibitors acted on blood stage replication and on exflagellation in similar concentrations. These data indicate that the PfCLKs have an important role during schizogony and are further crucial during parasite transmission from the human to the mosquito. These findings make them potential candidates as targets for antimalarials with transmission blocking properties.

## Supporting Information

Figure S1 **Alignment of kinase domains of the four PfCLKs with SRPK Sky1p of** ***Saccharomyces cerevisae*** **.**
(PDF)

Figure S2 **Co-localization and control IFAs.**
(PDF)

Figure S3 **Controls of immunoprecipitation assays.**
(PDF)

Figure S4 **The CaM-dependent kinase PfPKRP.**
(PDF)

Table S1 **Primers used for cloning.**
(PDF)

Table S2 **Chemical structures and antimalarial activities of the CLK inhibitors tested in this study.**
(PDF)

Table S3 **Phosphorylation sites identified in the SR proteins of** ***P. falciparum*** **blood stage schizonts.**
(PDF)

Methods S1 **Synthesis of CLK inhibitors (experimental and analytical details).**
(PDF)
