# Supplementary material for: Inhibition of the SR Protein-Phosphorylating CLK Kinases of Plasmodium falciparum Impairs Blood Stage Replication and Malaria Transmission
Source: PLoS One. 2014 Sep 4;9(9):e105732. doi: 10.1371/journal.pone.0105732 (PMC4154858; doi:10.1371/journal.pone.0105732)
Supplement: Figure S1 — Alignment of kinase domains of the four PfCLKs with SRPK Sky1p of Saccharomyces cerevisae . (PDF) [file pone.0105732.s001.pdf]

```
PF14_0431   ..................................................
PF14_0408   YKVVKVLSKTQFSTTLKCLNLLYKKVKTDTQVFLPYCHKYMKDDSEITHD
PF11_0156   ..................................................
PFC0105w    ..................................................
1how        ......................................DYRPGGFHPAFK

                    1        10        20
PF14_0431   ........FLVIRKMGDGTFGRVLLCQHIDNK.................K
PF14_0408   KKKNNYDKFVNLNTIKKKKNENYNRQHDIKNNLHDNKHQIINNKKKVEPK
PF11_0156   ........SVVCELVGKGVFSNVLKCYDMVNK.................I
PFC0105w    ........YRIEGKLGWGHFSTVWVATDLKSKPL...............K
1how        GEPYKDARYILVRKLGWGHFSTVWLAKDMVNN.................T

                 30        40        50
PF14_0431   YYAVKVVRNIKKYTRSAKIEADILKKIQNDDINN................
PF14_0408   YVCLKVMKNGKQFLDQGLLELMVLNILCNANTNNN...............
PF11_0156   PVAVKVIRDNDMMKKAAEKEISILKKLNQYDKDN................
PFC0105w    FVAIKIQKGSETYTESAKCEINYLNTVKVNSFDSSWVELKEQQRERLFHY
1how        HVAMKIVRGDKVYTEAAEDEIKLLQ..RVNDADN....TKEDSMG.....

                60        70        80        90       100
PF14_0431   ...NNIVKYHGKFMYYD....HMCLIFEPLGPSLYEIITRNNY.NGFHIE
PF14_0408   LSNKNIIQLYDSFYYKE....HLIIVTEYMQSDLYNYFIRKGK..LGTLG
PF11_0156   ..KRHIIRLLSSIKYKN....HLCLVFEWMWGNLRIALKKYGNGHGLNAT
PFC0105w    NMTKGVVSFIDSFEHKGPNGTHICMVFEFMGPNLLSLIKHYDY.KGIPLN
1how        ..ANHILKLLDHFNHKGPNGVHVVMVFEVLGENLLALIKKYEH.RGIPLI

                   110       120       130
PF14_0431   DIKLYCIEILKALNYLR.KMSLTHTDLKPENILLDDP.............
PF14_0408   QLQILTKNLLEGLAYIH.SKNLIHCDLKPENIMINMKKNKKNHEKGKYNK
PF11_0156   AVHCYTKQLFIALRHMR.KCRIMHADLKPDNILIN...............
PFC0105w    LVRKIATHVLIGMQYLHDVCKIIHSDIKPENVLVSPLTTIPKPKDYTKDK
1how        YVKQISKQLLLGLDYMHRRCGIIHTDIKPENVLM................
```

Fig. S1, page 1

```
PF14_0431   ..................................................
PF14_0408   ..................................................
PF11_0156   ..................................................
PFC0105w    TKKKKNINEPPYVKHKLRPSNSDPSLLTSYSNIHALQETLTRKPYHYNTY
1how        ..................................................

PF14_0431   ..................................................
PF14_0408   ..................................................
PF11_0156   ..................................................
PFC0105w    FLNNPEKYRDNKMNPYLHRLPNDCLKKIDQDDSDETEEEDDLSDVDQNKE
1how        ..................................................

PF14_0431   ..................................................
PF14_0408   ..................................................
PF11_0156   ..................................................
PFC0105w    QNKNQLEVNLPNNKYPNSNDVYKFFEKDINKFPIYCDMFNHLIHPEALRL
1how        ..................................................

PF14_0431   .............................................IQIYR
PF14_0408   ............................................INIYNT
PF11_0156   ..................................................
PFC0105w    HELYMKNKKNIDSNNTMNDLGNNQNSHKVVYINTEDGEYCIRPYDPSVYY
1how        ..................................................

          160       170       180       190       200
PF14_0431   TKSTGIKLIDFGCATFKSDY.HGSIINTRQYRAPEVILNLGWDVSSDMWS
PF14_0408   KQFDKIKIIDFNSCIYESDK.LEMYIQTRSYRSPEVLLQQNYDRKIDIWS
PF11_0156   .......VCDLGSASDISENEITSYLVSRFYRAPEIILGFRYDAQIDVWS
PFC0105w    HEKSCYKICDLGNSLWIDES.RYAEIQTRQYRAPEVILKSGFNETADIWS
1how        .......IADLGNACWYDEH.YTNSIQTREYRSPEVLLGAPWGCGADIWS
```

```
                210        220            230        240
PF14_0431   FGCVLAELYTGSLLFRTHE......HMEHLAMMESIIQPIPKNMLY....
PF14_0408   LGCILFEFLTKKILFDYQN......IYRFIYSIVSYIGPFPFYMINNCRI
PF11_0156   AAATVFELATGKILFPGKS......NNHMIKLMMEYKGKFSHKMIK....
PFC0105w    FACMVFELVTGDFLFNPQKGDRYDKNEEHLSFIIEVLGNIPKHMID....
1how        TACLIFELITGDFLFEPDEGHSYTKDDDHIAQIIELLGELPSYLLR....

                                              250       260
PF14_0431   ..............................EATKTNGSKYVN........
PF14_0408   PHIFTKHGLIILKKFTTDNMYENYIKEEQLNQEDDEEIVFNSNDFFRLNK
PF11_0156   ..............................GGQFYSQHFNENLDFLYVD
PFC0105w    ..............................AGYNSHKYFNK.........
1how        ..............................NGKYTRTFFN..........

                               270            280            290
PF14_0431   ......KDELKLAWPENASS.....INSIKHVKKCLP......LYKIIKH
PF14_0408   KDNILTKDLLKNKNPNTSTPRKRNNNNNEIYYDVCYPSDNLLKNNFQISD
PF11_0156   RDHYSKKEVVRVISDLRPTKNI...TCDLLEHQYWLKGNSPKMQFLKKKI
PFC0105w    .....NNYRLKNIRNIKKYG.....LYKILKYKYNLP.........EKEI
1how        .....SRGLLRNISKLKFWP.....LEDVLTEKYKFS.........KDEA

                      300       310       320
PF14_0431   ELFCDFLYSILQIDPTLRPSPAELLKHKFL....................
PF14_0408   TLFVDFLSSLLQIDPSKRCNAMEALKHPWL....................
PF11_0156   KQLGDLLEKCLILDPSKRYTPDQALQHPYL....................
PFC0105w    SPLCSFLLPMLSVDPQTRPSAYTMLQHPWL....................
1how        KEISDFLSPMLQLDPRKRADAGGLVNHPWLKDTLGMEEIRVPDRELYGSG

PF14_0431   ................
PF14_0408   ................
PF11_0156   ................
PFC0105w    ................
1how        SDIPGWFEEVRDHKRH
```

Fig. S1. Alignment of kinase domains of the four PfCLKs with SRPK Sky1p of *Saccharomyces cerevisae* (PDB ID; 1how). The amino acid residues shown in red indicate highly conserved sequences essential for the kinase activity. PF14_0431, PfCLK-1; PF14_0408; PfCLK-2; PF11_0156, PfCLK-3; PFC0105w, PfCLK-4.
