# Supplementary material for: Inhibition of the SR Protein-Phosphorylating CLK Kinases of Plasmodium falciparum Impairs Blood Stage Replication and Malaria Transmission
Source: PLoS One. 2014 Sep 4;9(9):e105732. doi: 10.1371/journal.pone.0105732 (PMC4154858; doi:10.1371/journal.pone.0105732)
Supplement: Figure S2 — Co-localization and control IFAs. (PDF) [file pone.0105732.s002.pdf]

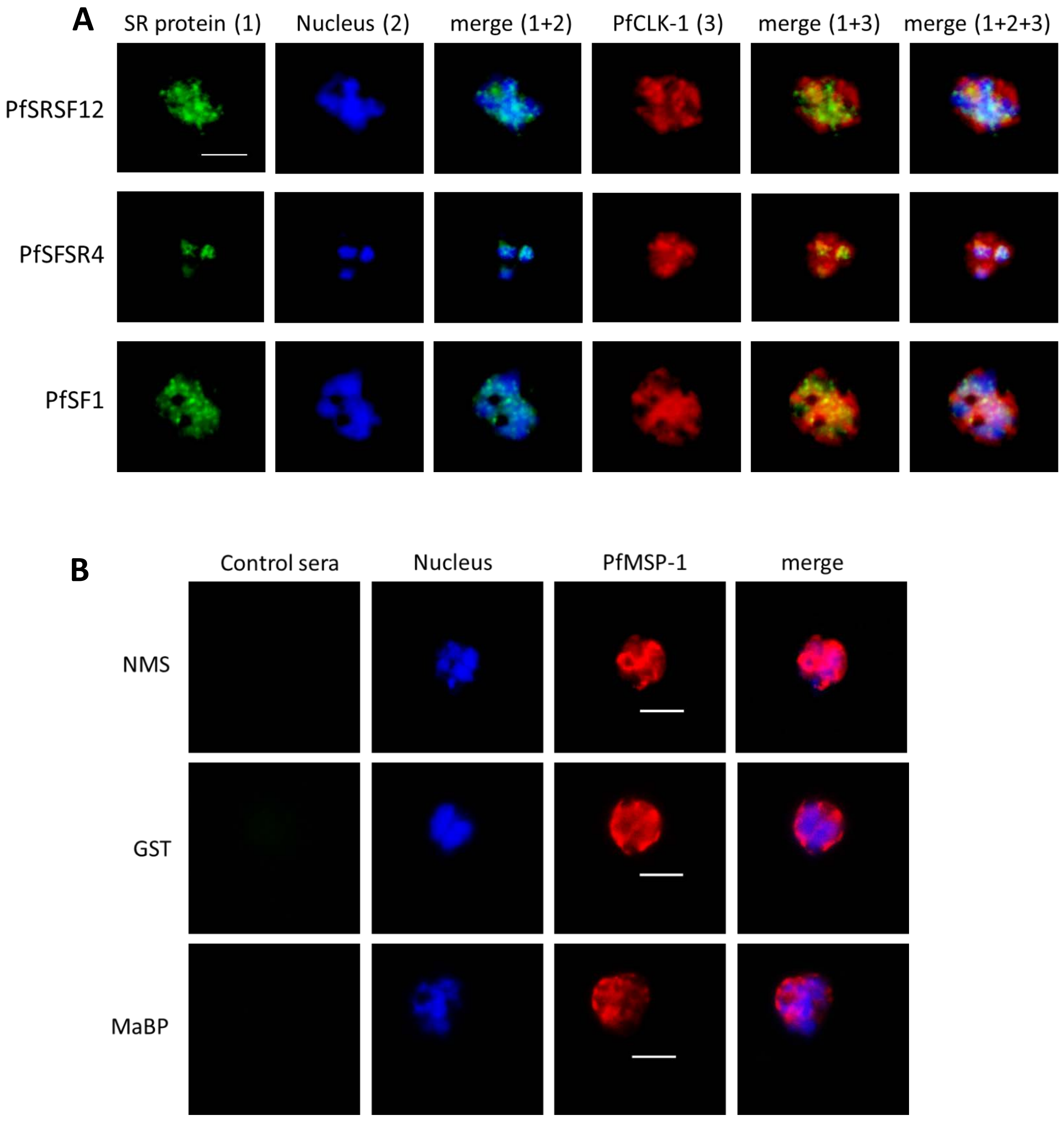

Fig. S2. Co-localization and control IFAs. A. Immunolabelling of PfCLK-1 with rabbit antisera (red) detected the kinase in the schizonts´ cytoplasm as well as in the nuclei, where it co-localizes with the three SR proteins (green). B. IFAs using sera of non-immunized mice (NMS) or of mouse antisera directed against the GST- or the MaBP-tag did not result in any labelling of the blood stage parasites (green). Schizonts were counterlabelled with rabbit antibodies against PfMSP-1 (red). All nuclei were highlighted by Hoechst staining (blue). Bar, 5 µm.

Fig. S2
