# Supplementary material for: Inhibition of the SR Protein-Phosphorylating CLK Kinases of Plasmodium falciparum Impairs Blood Stage Replication and Malaria Transmission
Source: PLoS One. 2014 Sep 4;9(9):e105732. doi: 10.1371/journal.pone.0105732 (PMC4154858; doi:10.1371/journal.pone.0105732)
Supplement: Figure S3 — Controls of immunoprecipitation assays. (PDF) [file pone.0105732.s003.pdf]

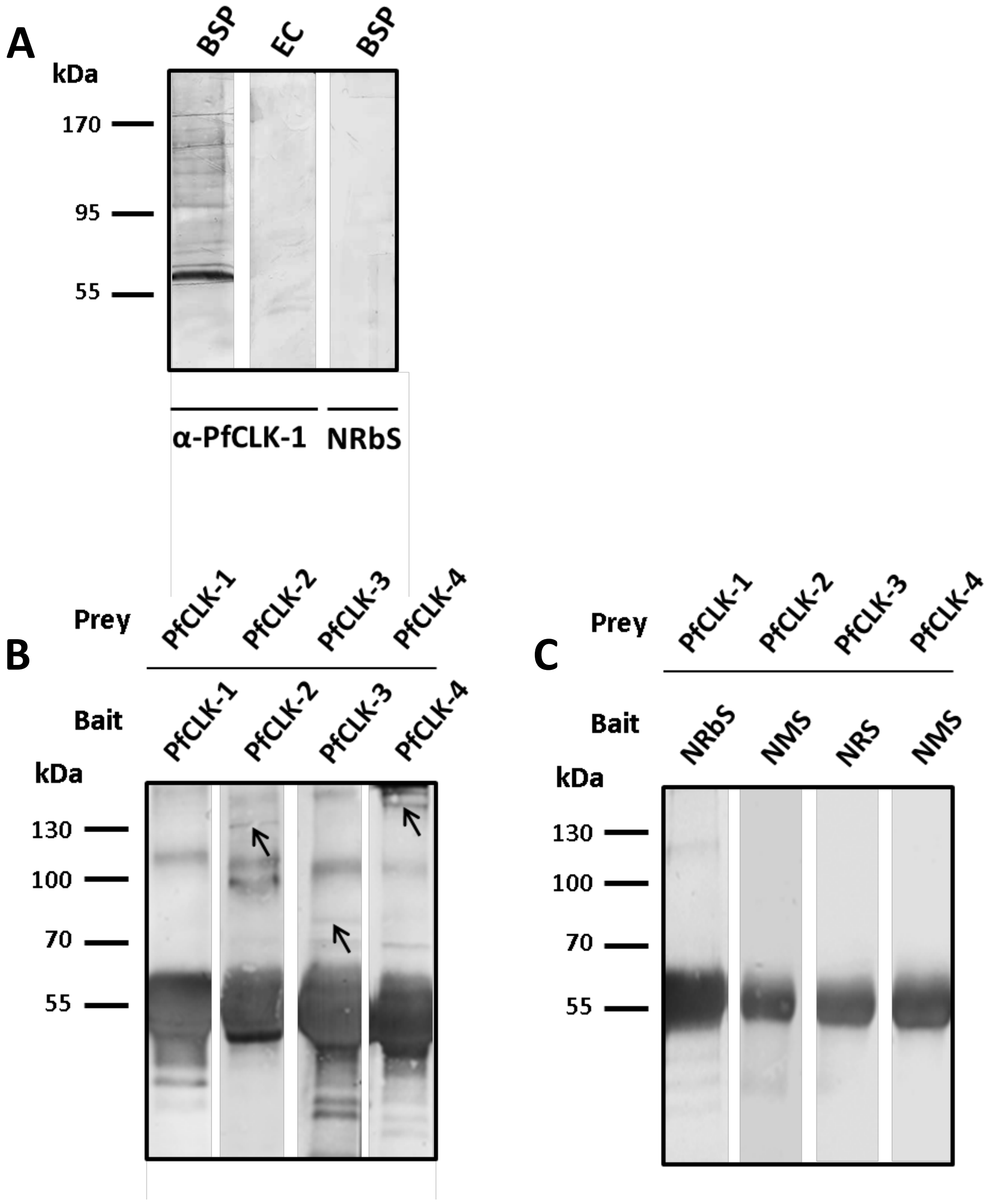


Fig. S3. Controls of immunoprecipitation assays. A. Western-blot analysis on lysates of blood stage parasites (BSP) using rabbit antisera against PfCLK-1 detected a processed 60 kDa band. No proteins were detected by the anti-PfCLK1 antisera in lysates of non-infected erythrocytes (EC). Immunoblotting of BSP with sera from non-immunized rabbit (NRbS) did not result in any labelling. B. Immunoprecipitation assays using PfCLK-specific antisera (bait) were performed and the precipitated proteins were immunoblotted using the same antisera (prey). Western blotting detected the precipitated PfCLK-2-4 proteins (arrows). An additional prominent protein band of approximately 55 kDa represents the heavy chain of the precipitating antibody. C. No protein bands were detected, when sera of non-immunized animals were used as bait .
