# Supplementary material for: Inhibition of the SR Protein-Phosphorylating CLK Kinases of Plasmodium falciparum Impairs Blood Stage Replication and Malaria Transmission
Source: PLoS One. 2014 Sep 4;9(9):e105732. doi: 10.1371/journal.pone.0105732 (PMC4154858; doi:10.1371/journal.pone.0105732)
Supplement: Figure S4 — The CaM-dependent kinase PfPKRP. (PDF) [file pone.0105732.s004.pdf]

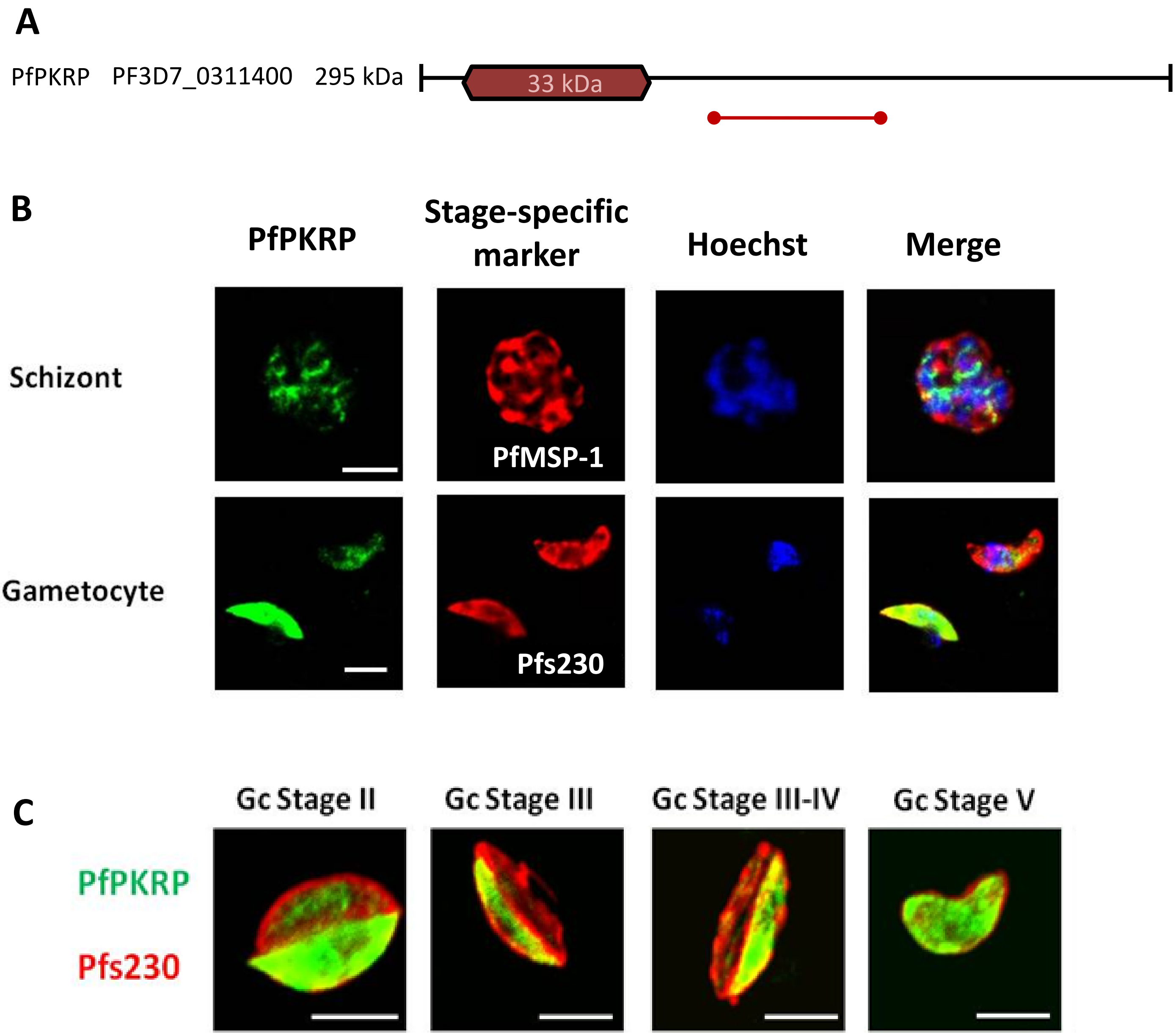

Figure S4. The calmodulin-dependent kinase PfPKRP. A. Domain structure of PfPKRP. The underlined region denotes the region of the recombinant protein. B, C. Expression of PfPKRP in the asexual blood and sexual stages of *P. falciparum*. IFAs were performed using mouse polyclonal antisera directed against PfPKRP and detected the kinase predominantly in gametocytes (Gc) of stages II-V (of strain NF54), while only a minor labelling was observed in schizonts (of gametocyte-less strain F12; in green). Asexual parasites were detected with rabbit antibodies against the merozoite surface protein PfMSP-1 and gametocytes were labelled with rabbit antibodies against Pfs230 (in red). Nuclei were highlighted by Hoechst 33342 staining (in blue). Bar, 5 µm.
