# Supplementary material for: Inhibition of the SR Protein-Phosphorylating CLK Kinases of Plasmodium falciparum Impairs Blood Stage Replication and Malaria Transmission
Source: PLoS One. 2014 Sep 4;9(9):e105732. doi: 10.1371/journal.pone.0105732 (PMC4154858; doi:10.1371/journal.pone.0105732)
Supplement: Table S1 — Primers used for cloning. (PDF) [file pone.0105732.s005.pdf]

Table S1. Primers used for recombinant protein expression.

**Protein**	**Forward primer**	**Reverse primer**	**Tag**	**MW**
PfCLK-4	5'- TA GGATCC TCC AAT AAC AGC AAC AGT -3'	5'- TA CCCGGG **TTA** TTT GGT AATC CCT TCCG CTTT -3'	GST-tag	77 kDa
PfSFRS4	5´- AA GAATTC GAT GAT GGT GTT GGT CCA-3´	5´- TA GTCGAC **TTA** CAT TTT CAT CTG CTG CAT TAG-3´	MaBP-tag	65 kDa
PfSRSF12	5´- AA GAATTC ATG AAA AAG TTA ATT AAT TGT GGC-3´	5´-TT GCGGCCGC **TTA** ATT TAG TTC CTT TGG AGA-3´	MaBP-tag	73 kDa
PfSF-1 N-terminal	5´- GCG TA GGATCC GAA CAC GAT GGT GAA GAT GAC GGT -3	5´- TACGC GTCGAC **TTA** CCT ATC TCT TTC TCT TTC TCT ATC TCT -3´	MaBP-tag	95 kDa
PfSF-1 C-terminal	5´- GCGTA GGATCC GAA GAA ATG GAA GAA GCA AAA AGG GAT-3´	5´-TA GTCGAC **TTA** CTT TGG AAG ACA AGT CAT ATC CCA TAC ATC-3´	MaPB-tag	86 kDa
PfPKRP	5´- TA GAATTC CAT GTA TTT GTC TCT TCC TCT -3´	5´- TA CCCGGG **TTA** ACT ACC GTC CAC CTT ACT AAC -3´	GST-tag	79 kDa

Restriction sites underlined; stop codon in bold; MW, molecular weight.
