# Supplementary material for: Inhibition of the SR Protein-Phosphorylating CLK Kinases of Plasmodium falciparum Impairs Blood Stage Replication and Malaria Transmission
Source: PLoS One. 2014 Sep 4;9(9):e105732. doi: 10.1371/journal.pone.0105732 (PMC4154858; doi:10.1371/journal.pone.0105732)
Supplement: Table S2 — Chemical structures and antimalarial activities of the CLK inhibitors tested in this study. (PDF) [file pone.0105732.s006.pdf]

Table S2. Chemical structures and antimalarial activities of the CLK inhibitors tested in this study.

Substance name	structure	IC_{50} Malstat assay [µM]				IC_{50} mean ± SEM [µM]
**Aminopyrimidines**						
C117	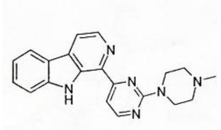	5.5	9.4		-	**7.5 ± 2.76**
C81	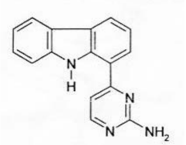	36.1	77.8	40.2	-	**51.4 ± 22.98**
C-129	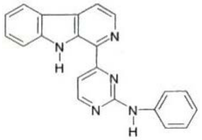	>100	>100	-	-	**>100**
C-666-42-72	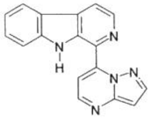	>100	-	-	-	**>100**
C-667	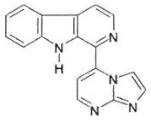	>100	>100	-	-	**>100**
EK-28	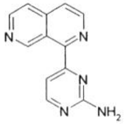	>100	-	-	-	**>100**
Kast-24	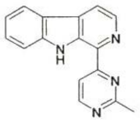	30.7	58.9	42.7	-	**44.1 ± 14.15**

Kast-25	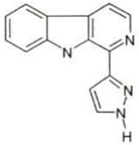	99.7	75.8	-	-	**87.8 ± 16.90**
Kast-27	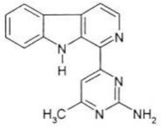	>100	>100	-	-	**>100**
Kast-50	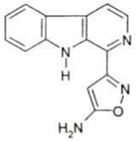	11.0	9.9	19.3	6.7	**11.7 ± 5.37**
Kast-73	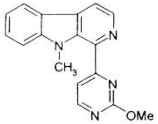	>100	>100	-	-	**>100**
Kast180-HCl	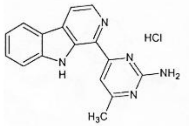	>100	>100	-	-	**>100**
Puzik-V8	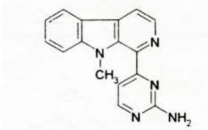	>100	>100	-	-	**>100**
Puzik-V12	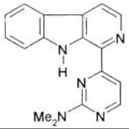	16.4	28.6	37.7	-	**27.6 ± 10.69**
Puzik-V16	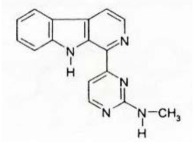	55.7	25.3	-	-	**40.5 ± 21.50**

Puzik-V23.1	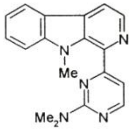	39.1	32.9	34.9	-	**35.6 ± 3.16**
gea_27	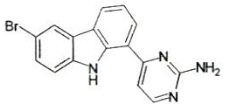	5.4	4.9	-	-	**5.2 ± 0.35**
gea_50	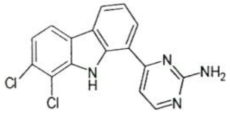	>100		-	-	**>100**
gea_70	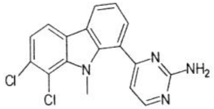	>100	>100	-	-	**>100**
gea_75	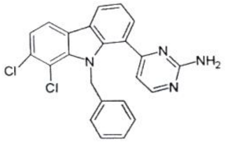	3.6	33.8	-	-	**18.7 ± 21.35**
**Oxo-β-carbolines**						
KH-CARB13xHCl	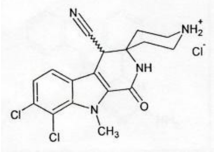	6.4	2.0	4.2	5.0	**4.4 ± 1.84**
Pohl-2	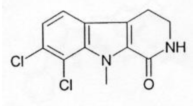	121.1	61.8	72.8	-	**85.2 ± 31.54**
Pohl-17	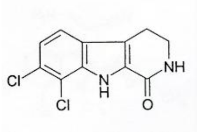	>100	>100	-	-	**>100**

KH-CARB1	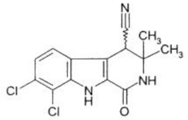	72.2	119.1	-	-	**95.7 ± 33.16**
KH-CARB8	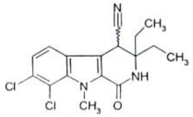	59.5	49.5	-	-	**54.5 ± 7.07**
KH-CARB3A	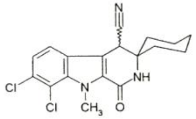	>100	>100	-	-	**>100**
KH-CARB3B	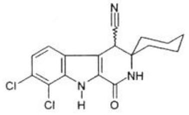	>100	>100	-	-	**>100**
KH-CARB7	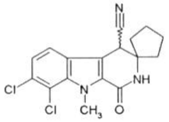	>100	>100	-	-	**>100**
KH-CARB9	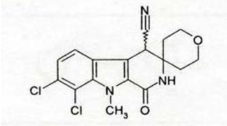	>100	>100	-	-	**>100**
KH-CARB10	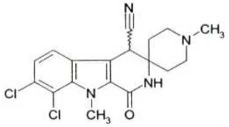	9.2	9.1	4.3	6.1	**7.2 ± 2.40**
KH-CARB11	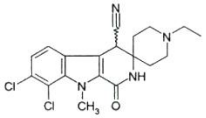	7.1	6.4	5.0	-	**6.1 ± 1.09**
KH-CARB2	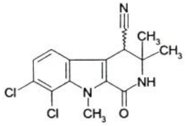	142.4	63.2	-	-	**102.8 ± 56.00**

KH-DTCMA	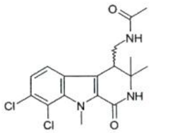	8.1	23.5	-	-	**15.8 ± 10.92**
KH-CARB6	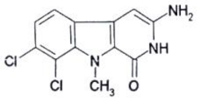	76.3	60.3	-	-	**68.3 ± 11.31**
KH-AMTC	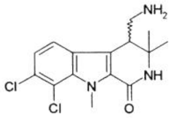	6.0	11.9	-	-	**9.0 ± 4.17**
KH-CB5	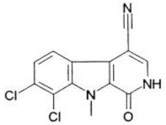	>100	-	-	-	**>100**
KH-CB19T	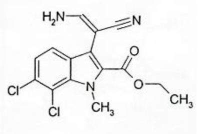	>100	-	-	-	**>100**
NIH85	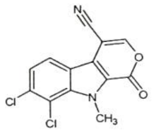	>100	>100	-	-	**>100**
AR7	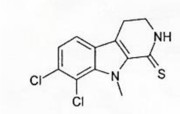	>100	>100	-	-	**>100**
**Tetramic acids**						
KH-HP05	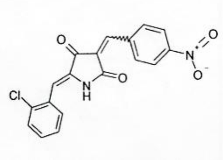	36.0	46.8	41.2	-	**41.3 ± 5.40**

KH-HP11	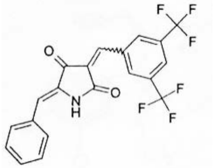	29.3	43.8	43.7	-	**38.9 ± 8.34**
KH-HP01	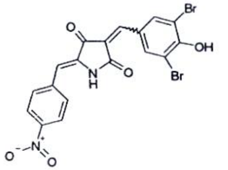	>100	>100	-	-	**>100**
KH-HP02	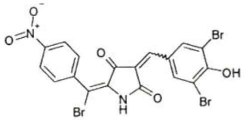	32.6	47.4	-	-	**40.0 ± 10.47**
KH-HP03	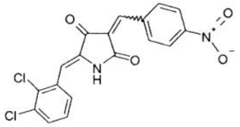	90.4	52.4	-	-	**71.4 ± 26.87**
KH-HP04	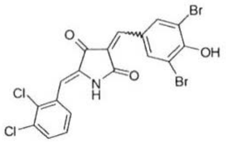	>100	-	-	-	**>100**
KH-HP06	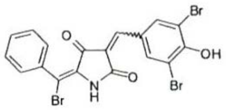	>100	>100	-	-	**>100**
KH-HP07	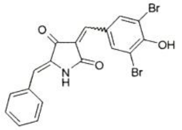	>100	-	-	-	**>100**

KH-HP08	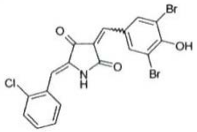	56.8	57.3	-	-	**57.1 ± 0.35**
KH-HP09	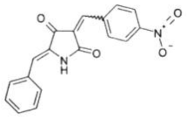	37.4	66.1	-	-	**51.8 ± 20.29**
**Carbolines/Carbazoles**						
CS-14	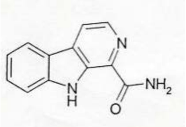	59.6	29.9	97.2	79.0	**66.4 ± 28.79**
CS02	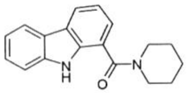	>100	>100	-	-	**>100**
CS04	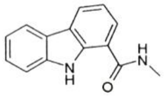	>100	-	-	-	**>100**
CS06	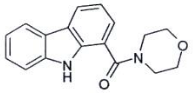	>100	>100	-	-	**>100**
CS07	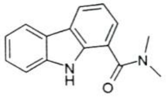	>100	>100	-	-	**>100**
gea_11	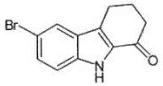	>100	-	-	-	**>100**
gea_49	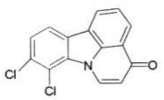	25.8	64.0	-	-	**44.9 ± 27.01**
gea_55	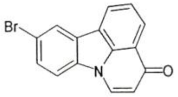	67.2	64.3	-	-	**65.6 ± 2.05**

**Indoles/Benzothiazoles**						
NIH11	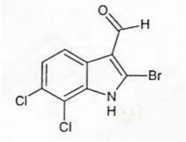	23.0	55.0	17.7	-	**31.9 ± 20.18**
NIH16	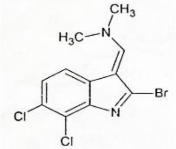	50.5	91.3	34.4	-	**58.7 ± 29.33**
KH-CM16	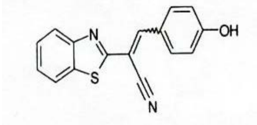	78.2	130.8	-	-	**104.5 ± 37.19**
NIH08	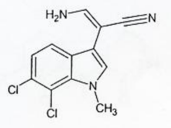	>100	>100	-	-	**>100**
NIH39	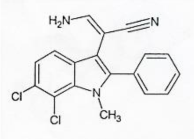	>100	-	-	-	**>100**
NIH54	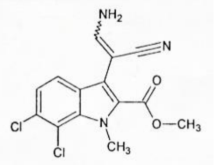	>100	>100	-	-	**>100**
**Antiseptic**						
Chlorhexidine	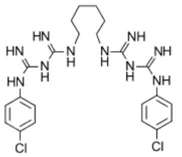	0.4	0.8	0.7	-	**0.6 ± 0.20**
