# Supplementary material for: Inhibition of the SR Protein-Phosphorylating CLK Kinases of Plasmodium falciparum Impairs Blood Stage Replication and Malaria Transmission
Source: PLoS One. 2014 Sep 4;9(9):e105732. doi: 10.1371/journal.pone.0105732 (PMC4154858; doi:10.1371/journal.pone.0105732)
Supplement: Table S3 — Phosphorylation sites identified in the SR proteins of P. falciparum blood stage schizonts. (PDF) [file pone.0105732.s007.pdf]

Table S3. Phosphorylation sites identified in the SR proteins of *P. falciparum* blood stage schizonts.

Gene-ID	Name	1	2	3	4
**PF3D7_0503300**	**PfSRSF12**	S24	T23	S24	S24
		S258	S24	S26	S26
			S26		S131
			S169		S132
			S255		
			S258		
**PF3D7_1022400**	**PfSFRS4**	S207	S8	S207	S197
		S213	S10	S210	S213
		S245	S12	S213	S268
		S438	S50	S310	S271
			S51	S345	S368
			S197	S349	S369
			S207	S368	S388
			S213	S432	S392
			S270	S438	S395
			S271	S537	S537
			S280		
			S390		
			S392		
			S438		
			S537		
**PF3D7_1321700**	**PfSF-1**	S19	S28	S28	None
			S29	T693	
			S175		
			T693		

S, serine; T, tyrosine.

1 Solyakov et al. (2011) Global kinomic and phospho-proteomic analyses of the human malaria parasite Plasmodium falciparum. Nat Commun 2: 565.
2 Treeck et al. (2011) The phosphoproteomes of Plasmodium falciparum and Toxoplasma gondii reveal unusual adaptations within and beyond the parasites' boundaries. Cell Host Microbe 10: 410-419.
3 Lasonder et al. (2012) The Plasmodium falciparum schizont phosphoproteome reveals extensive phosphatidylinositol and cAMP-protein kinase A signaling. J Proteome Res 11: 5323-5337.
4 Pease et al. (2013) Global analysis of protein expression and phosphorylation of three stages of Plasmodium falciparum intraerythrocytic development. J Proteome Res 12: 4028-4045.
