# Supplementary material for: Inhibition of the SR Protein-Phosphorylating CLK Kinases of Plasmodium falciparum Impairs Blood Stage Replication and Malaria Transmission
Source: PLoS One. 2014 Sep 4;9(9):e105732. doi: 10.1371/journal.pone.0105732 (PMC4154858; doi:10.1371/journal.pone.0105732)
Supplement: Methods S1 — Synthesis of CLK inhibitors (experimental and analytical details). (PDF) [file pone.0105732.s008.pdf]

Methods S1. Synthesis of CLK inhibitors (experimental and analytical details)

1-[2-(4-Methylpiperazin-1-yl)-pyrimidin-4-yl]-β-carboline (C-117)

A solution of 428 mg (2.03 mmol) of 1-acetyl-β-carboline (1) in 10 ml anhydrous DMF was treated with 840 µl (4.07 mmol) Bredereck's reagent and refluxed for 1 h under nitrogen. Then 2.33 g (6.10 mmol) 4-methylpiperazine-1-carboxamidinium sulfate and 562 mg (4.07 mmol) potassium carbonate were added, and the mixture was refluxed for another 18 h. After cooling to ambient temp. 20 ml saturated sodium bicarbonate solution were added, and the mixture was extracted with ethyl acetate (3 x 25 ml). The combined organic layers dried over sodium sulfate, evaporated, and the residue purified by column chromatography on silica gel (eluent: cyclohexane/ethyl acetate/ ethyl dimethylamine 9:9:2) to give 410 mg (58%) of C-117 ($C_{20}H_{20}N_6$, Mr = 344.42 g/mol) as a yellow solid, mp 135 – 136 °C.

^{1}H NMR (500 MHz, $CDCl_3$): δ (ppm) = 10.72 (s, 1 H, N-H), 8.54 (d, J = 5.1 Hz, 1 H, 3-H), 8.53 (d, J = 5.1 Hz, 1 H, 6'-H), 8.15 (dd, J = 0.9 Hz, 7.9 Hz, 1 H, 5-H), 8.02 (dd, J = 5.1 Hz, 0.4 Hz, 1 H, 4-H), 7.82 (d, J = 5.1 Hz, 1 H, 5'-H), 7.58 (dt, J = 1.1 Hz, 7.6 Hz, 1 H, 7-H), 7.51 (ddd, J = 0.9 Hz, 1.1 Hz, 6.4 Hz, 1 H, 8-H), 7.31 (dt, J = 0.9 Hz, 7.5 Hz, 1 H, 6-H), 4.01 (t, J = 5.1 Hz, 4 H, 2''-H, 6''-H), 2.62 (t, J = 5.2 Hz, 4 H, 3''-H, 5''-H), 2.41 (s, 3 H, N-CH_3). ^{13}C NMR (125 MHz, $CDCl_3$): δ (ppm) = 165.2 (C-4'), 161.9 (C-2'), 158.6 (C-6'), 140.3 (C-8a), 138.7 (C-3), 136.8 (C-1), 135.0 (C-9a), 130.8 (C-4a), 128.7 (C-7), 121.9 (C-5), 121.0 (C-4b), 120.2 (C-6), 116.5 (C-4), 111.6 (C-8), 107.0 (C-5'), 54.9 (C-3'', C-5''), 46.3 (N-CH_3), 44.4 (C-2'', C-6''). IR (KBr): (cm^{-1}) = 3397, 2926, 2851, 2799, 1565, 1487, 1436, 1340, 1213, 1158, 1069, 989, 832, 748. MS(EI): m/z (rel. int. in %) = 344 $[M]^{+\bullet}$ (61); 287 (17); 274 (100); 262 (10).

1-{6-Bromo-9-[(2-(trimethylsilyl)ethoxy)methyl]-9H-carbazol-1-yl}ethanone (3)

A solution of 922 mg (3.20 mmol) 1-(6-bromo-9H-carbazol-1-yl)ethanone (2) in 30 ml anhydrous THF was added slowly to an ice-cooled suspension of 194 mg (4.85 mmol) sodium hydride (60 % in mineral oil) in 10 ml anhydrous THF, and the mixture was stirred at 0 °C for 30 min. Then 0.92 ml (4.8 mmol) 2-(trimethylsilyl)ethoxymethyl chloride (SEM-Cl) were added, the mixture was stirred at ambient temp. for 22 h and then poured on ice-water. The organic layer was separated, washed with satd. sodium bicarbonate solution and brine, dried over sodium sulfate, and evaporated. The residue was purified by column chromatography on silica gel (eluent: cyclohexane/ethyl acetate 4:1) to give 900 mg (67%) of 3 ($C_{20}H_{24}BrNO_2Si$, Mr = 418.40 g/mol) as yellow crystals, mp 74 – 76 °C.

^{1}H NMR (500 MHz, CD_2Cl_2): δ (ppm) = 8.23 (d, J = 2.0 Hz, 1H, 5-H), 8.20 (dd, J = 7.8 Hz, 1.2 Hz, 1H, 4-H), 7.73 (dd, J = 7.6 Hz, 1.2 Hz, 1H, 2-H), 7.59 (dd, J = 8.7 Hz, 2.0 Hz, 1H, 7-H), 7.44 (d, J = 8.7 Hz, 1H, 8-H), 7.34 (t, J = 7.6 Hz, 1H, 3-H), 5.68 (s, 2H, 1´´-H), 3.08 (t, J = 8.3 Hz, 2H, 2´´-H), 2.71 (s, 3H, 2´-H), 0.70 (t, J = 8.3 Hz, 2H, 3´´-H), -0.14 (s, 9H, 4´´-H). ^{13}C NMR (125 MHz, CD_2Cl_2): δ (ppm) = 201.1 (C=O), 141.1 (C-8a), 135.7 (C-9a), 129.5 (C-7), 127.3 (C-1), 126.9 (C-2), 125.1 (C-4a), 124.8 (C-4b), 123.8 (C-4), 123.3 (C-5), 119.8 (C-3), 113.4 (C-6), 111.7 (C-8), 73.6 (C-1´´), 65.4 (C-2´´), 29.8 (C-2´), 18.1 (C-3´´), -1.6 (C-4´´). IR (KBr): (cm^{-1}) = 3448, 2951, 2886, 2362, 1664, 1578, 1492, 1458, 1264, 1247, 1203, 1137, 1068, 993, 967, 860, 837, 805, 733, 610. MS(EI): m/z (rel. int. in %) = 419 [$M^{+\bullet}$] (5), 417 (5), 390 (5), 346 (40), 344 (35), 301 (10), 272 (10), 102 (10), 73 (100). HR-MS (EI): m/z = 417.0753 (calcd. for $C_{20}H_{24}BrNO_2Si$: 417.0760).

4-{6-Bromo-9-[(2-(trimethylsilyl)ethoxy)methyl]-9H-carbazol-1-yl}pyrimidin-2-amine (4)

A solution of 900 mg (2.15 mmol) of methyl ketone 3 in 25 ml anhydrous DMF was treated with 885 µL (4.23 mmol) Bredereck´s reagent and refluxed for 1 h under nitrogen. Then 1.2 g (6.5 mmol) guanidinium carbonate and 580 mg (4.20 mmol) potassium carbonate were added, and the mixture was refluxed for another 18 h. After cooling to ambient temp. 25 ml saturated sodium bicarbonate solution and 25 ml brine were added, and the mixture was extracted with ethyl acetate (3 x 30 ml). The combined organic layers dried over sodium sulfate, evaporated, and the residue purified by column chromatography on silica gel (eluent: dichloromethane/ethanol 9:1) to give 960 mg (95%) of 4 ($C_{22}H_{25}BrN_4OSi$, Mr = 469.45 g/mol) as a pale yellow solid, mp 128 – 129 °C.

^{1}H NMR (500 MHz, CD_2Cl_2): δ (ppm) = 8.39 (d, J = 5.0 Hz, 1H, 6´-H), 8.24 (d, J = 2.0 Hz, 1H, 5-H), 8.13 (dd, J = 7.7 Hz, 1.3 Hz, 1H, 4-H), 7.57 (dd, J = 8.7 Hz, 2.0 Hz, 1H, 7-H), 7.47 – 7.42 (m, 2H, 2-H, 8-H), 7.34 (t, J = 7.6 Hz, 1H, 3-H), 6.94 (d, J = 5.0 Hz, 1H, 5´-H), 5.53 (s, 2H, 1´´-H), 3.03 (t, J = 8.2 Hz, 2H, 2´´-H), 0.63 (t, J = 8.2 Hz, 2H, 3´´-H), -0.17 (s, 9H, 4´´-H). ^{13}C NMR (125 MHz, CD_2Cl_2): δ (ppm) = 167.8 (C-4´), 163.0 (C-2´), 159.0 (C-6´), 140.8 (C-8a), 137.5 (C-9a), 129.3 (C-2, C-7), 125.4 (C-4b), 124.8 (C-1), 124.7 (C-4a), 123.2 (C-5), 121.8 (C-4), 120.5 (C-3), 113.4 (C-6), 112.2 (C-5´, C-8), 74.2 (C-1´´), 65.6 (C-2´´), 18.0 (C-3´´), -1.5 (C-4´´). IR (KBr): (cm^{-1}) = 3330, 2923, 2361, 1736, 1618, 1571, 1456, 1249, 1214, 1072, 858, 837, 800, 748. MS(EI): m/z (rel. int. in %) = 470 [$M^{+\bullet}$] (5), 468 (5), 397 (25), 395 (20), 369 (65), 367 (65), 353 (15), 351 (15), 341 (30), 339 (30), 272 (10), 260 (10), 73 (100). HR-MS (EI): m/z = 468.1010 (calcd. for $C_{22}H_{25}BrN_4OSi$: 468.0981).

4-(6-Bromo-9H-carbazol-1-yl)pyrimidin-2-amine (gea-27)

A solution of 1.03 g (2.19 mmol) SEM-carbazole 4 in 50 ml THF was treated with 4 ml hydrofluoric acid (40 % in water) and stirred at ambient temp. for 15 h. The mixture was neutralized by careful addition of satd. sodium bicarbonate solution, 20 ml brine were added, followed by extraction with ethyl acetate (3 x 40 ml). The combined organic layers dried over sodium sulfate, evaporated, and the residue purified by column chromatography on silica gel (eluent: (cyclohexane/ethyl acetate 1:1, then dichloromethane/ethanol 1:1) to give 485 mg (65%) of gea-27 ($C_{16}H_{11}BrN_4$, Mr = 339.19 g/mol) as a yellow solid, mp 237 – 239 °C.

^{1}H NMR (500 MHz, DMSO-d_6): δ (ppm) = 12.04 (br s, 1H, 9-NH), 8.47 (d, J = 2.0 Hz, 1H, 5-H), 8.38 (d, J = 7.7 Hz, 1H, 4-H), 8.37 (d, J = 5.4 Hz, 1H, 6´-H), 8.20 (d, J = 7.8 Hz, 1H, 2-H), 7.68 (d, J = 8.6 Hz, 1H, 8-H), 7.62 (dd, J = 8.6 Hz, 2.0 Hz, 1H, 7-H), 7.41 (d, J = 5.5 Hz, 1H, 5´-H), 7.32 (t, J = 7.7 Hz, 1H, 3-H). ^{13}C NMR (100 MHz, CD_2Cl_2): δ (ppm) = 163.3 (C-4´), 162.8 (C-2´), 158.2 (C-6´), 137.9 (C-8a), 137.6 (C-9a), 128.0 (C-7), 124.6 (C-2), 123.2 (C-4b), 123.1 (C-4), 122.8 (C-4a), 122.4 (C-5), 118.4 (C-3), 117.9 (C-1), 113.1 (C-8), 110.7 (C-6), 104.4 (C-5´). IR (KBr): (cm^{-1}) = 3424, 3352, 2362, 1613, 1573, 1542, 1502, 1459, 1351, 1268, 1222, 1050, 804, 791, 744, 631, 597. MS(EI): m/z (rel. int. in %) = 340 [$M^{+\bullet}$] (100), 338 (100), 324 (25), 322 (25), 258 (10), 130 (15), 57 (15). HR-MS (EI): m/z = 338.0139 (calcd. for $C_{16}H_{11}BrN_4$: 338.0167).
